# Operando time-resolved X-ray absorption spectroscopy reveals the chemical nature enabling highly selective CO_2_ reduction

**DOI:** 10.1038/s41467-020-17231-3

**Published:** 2020-07-14

**Authors:** Sheng-Chih Lin, Chun-Chih Chang, Shih-Yun Chiu, Hsiao-Tien Pai, Tzu-Yu Liao, Chia-Shuo Hsu, Wei-Hung Chiang, Ming-Kang Tsai, Hao Ming Chen

**Affiliations:** 1 Department of Chemistry, National Taiwan University, Taipei, 10617 Taiwan; 2 Department of Chemical and Material Engineering, Chinese Culture University, Taipei, 11114 Taiwan; 3 Department of Chemistry, National Taiwan Normal University, Taipei, 11677 Taiwan; 4 Department of Chemical Engineering, National Taiwan University of Science and Technology, Taipei, 10607 Taiwan; 5 National Synchrotron Radiation Research Center, Hsinchu, 30076 Taiwan

**Keywords:** Electrochemistry, Energy, Materials chemistry

## Abstract

Copper electrocatalysts have been shown to selectively reduce carbon dioxide to hydrocarbons. Nevertheless, the absence of a systematic study based on time-resolved spectroscopy renders the functional agent—either metallic or oxidative Copper—for the selectivity still undecidable. Herein, we develop an operando seconds-resolved X-ray absorption spectroscopy to uncover the chemical state evolution of working catalysts. An oxide-derived Copper electrocatalyst is employed as a model catalyst to offer scientific insights into the roles metal states serve in carbon dioxide reduction reaction (CO_2_RR). Using a potential switching approach, the model catalyst can achieve a steady chemical state of half-Cu(0)-and-half-Cu(I) and selectively produce asymmetric C_2_ products - C_2_H_5_OH. Furthermore, a theoretical analysis reveals that a surface composed of Cu-Cu(I) ensembles can have dual carbon monoxide molecules coupled asymmetrically, which potentially enhances the catalyst’s CO_2_RR product selectivity toward C_2_ products. Our results offer understandings of the fundamental chemical states and insights to the establishment of selective CO_2_RR.

## Abstract (web summary)

A systematic time-resolved study can provide key insights on selective carbon dioxide electro-reduction. Here, the authors report operando seconds-resolved X-ray absorption spectroscopy to uncover the chemical state evolution of working catalysts in a carbon dioxide electroreduction process.

## Introduction

Of versatility, energy efficiency, and cost effectiveness, electrochemical CO_2_ reduction (eCO_2_RR) has been deemed a potential strategy to mitigate excessive anthropogenic CO_2_ emissions^1–8^. From a variety of catalysts for eCO_2_RR, those copper-based have drawn much attention^1,6^, since Cu is the one which can electrochemically reduce CO_2_ molecules to economically favorable and energy-dense C_2_ products, such as C_2_H_4_ and C_2_H_5_OH^5,9^. However, the typically low product selectivity hinders the catalysts from industry applications. There, accordingly, have been numerous studies on improving the selectivity by means of nanostructuring^10,11^, bimetallic systems^12–14^, and oxidative modifications^15–18^. Among them oxidative modifications have received considerable attention, since they could significantly improve the selectivity of Cu-based catalysts toward valuable C_2_ products.

Nevertheless, despite showing improvements, these oxidative modifications still cannot meet the decent selectivity for industry applications, 90% as Jouny et al. pointed out^5^. It has been noticed that these studies, whether knowingly or otherwise, suggested that Cu(I) may play a critical role in improving the selectivity toward C_2_ products, but the chemical state of Cu in the catalysts were varied drastically during electrochemical reduction by using a chronoamperometry^15,18^. This ineffective control of the chemical compositions and/or chemical states leads to the resulting low selectivity toward C_2_ products, and a fair speculation could be that a stable chemical composition of Cu species during electrolysis is indispensable to enhance the related C_2_ product selectivity. For instance, Nogami et al. showed that an oxidation process stimulated by switching applied potentials in corresponding time intervals could bring an improvement in the product selectivity in their eCO_2_RR system^19^. Recent studies also agreed with the integrated oxidation process for improving CO_2_RR product selectivity^20–23^. For example, the employment of pulses voltage has been revealed to enhance the selectivity toward the C_2_ products on polycrystalline Cu^21,23,24^. Recently, we have justified the existence of another competing reaction (i.e., the spontaneous oxidation of Cu(0) in aqueous electrolyte) that significantly governs the chemical state of active centers of Cu, establishing a strong correlation between the chemical state under reaction conditions and the CO_2_RR product profile^25^. A similar effect has been also revealed in the case of Fe single atom catalyst, in which the reduction activity toward CO is evidently dominated by the chemical state of metal centers^26^.

However, some studies brought a different perspective that the active catalyst is metallic copper because no significant concentration of residual oxide was detected in the oxide-derived samples; ambient pressure X-ray photoelectron spectroscopy (XPS) and electron energy loss spectroscopy (EELS) proved the absence of residual copper oxide in the reduced electrocatalyst^27,28^. Regarding this controversial issues, we have to point out a paramount subject concerning a significant mismatch in time-scale of acquiring spectrum. For conventional X-ray absorption spectroscopy (XAS), the spectroscopic acquiring time is approximately several 10 min (normally 20–30 min). In the cases of XAS and EELS, both of them would require a time-scale in minutes at least for spectrum acquisition. Notably, the chemical state of metal centers could reach a steady state in a few minutes under the eCO_2_RR; as revealed by in situ Raman, the CuO_x_ precursor would achieve a steady-state condition due to the co-occurrence of electrochemical reduction and of spontaneous oxidation caused by the trace oxidant in the electrolyte^25^. Consequently, a time-resolved identification of the characteristics in chemical state of metal centers should be highly desirable, especially for a time-scale of few seconds for revealing the real dominated factor in CO_2_ reduction electrocatalysis.

**Fig. 1:**
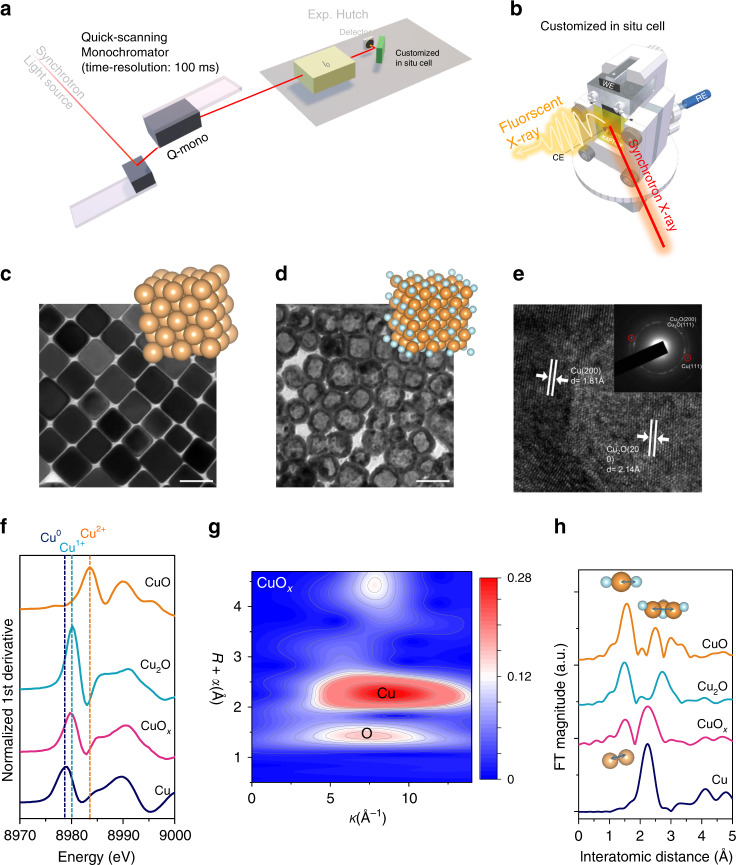
Experimental setup and catalyst characterization. **a** The schematic diagram of the setup of operando time-resolved XAS experiments. **b** The cartoon representation of customized operando XAS cell. **c** TEM image of Cu nanocubes. **d** TEM image of CuO_x_. Scale bars are 50 nm. **e** HR-TEM image and SAED pattern (inset) of CuO_x_. **f** First derivatives of the Cu K-edge XANES spectra of CuO_x_ and of commercial Cu, Cu_2_O, and CuO as references. **g**, **h** Wavelet transform-EXAFS of as-prepared CuO_x_ and the references.

Thus far, there have been various types of time-resolved XAS developed. For example, the fixed energy X-ray absorption voltammetry (FEXRAV) has demonstrated its capability to provide valency information of a material under time-dependent electrochemical working conditions^29–31^. Another technique in the time-resolved XAS family is energy-dispersive XAS (EDXAS). The parallel acquisition of all data points in a XAS spectrum using EDXAS allows its time resolution down to the level of ms and, if possible, to ps^32^. While the FEXRAV and the EDXAS are of time resolution, certain drawbacks make both of them possibly not suitable for the present study. In the case of the FEXRAV, it fails to offer the information regarding chemical surroundings, which has been reported to influence the selectivity of a catalyst^33,34^ and normally can be derived using EXAFS spectroscopy. These technical limitations can be well removed by using quick-XAS. The basic setup for quick-XAS is shown in Fig. 1a. Via a quick-scanning monochromator, an acquisition time of a single XAS spectrum, including X-ray absorption near edge structure (XANES) and extended X-ray absorption structure (EXAFS), can be cut down to a few seconds, reaching seconds-resolution. Moreover, its sequential detection mode also renders the operando detection achievable. Overall, the advantage of data collection and interpretation makes quick-XAS (TR-XAS hereafter) the time-resolved technique of choice in present study.

Herein, utilizing TR-XAS with a small X-ray incident angle, we develop an operando methodology to realize the seconds-resolved near-surface investigations of materials under CO_2_RR working conditions (Fig. 1a, b). This allows us to in situ characterize the chemical nature of catalytic surfaces. An oxide-derived Cu electrocatalyst with great potentials to produce hydrocarbons is employed as a model to provide scientific insights to the roles of metal states in CO_2_RR. We can conclude that chemical state of copper ions can reach a steady-state in ~5 min toward the metallic Cu(0) under a conventional chronoamperometry. Most interestingly, by utilizing a potential switching approach, the Cu(I)/Cu(0) redox could achieve a steady state of half-and-half during a cathodic CO_2_RR electrolysis, producing an asymmetric C_2_ product – C_2_H_5_OH – with fairly high selectivity in a wide potential range. Furthermore, a theoretical computation revealed that a surface composed of Cu–Cu(I) ensembles could have dual CO molecules coupled asymmetrically, which potentially enhanced the catalyst’s CO_2_RR product selectivity toward C_2_ products^35^. Operando TR-XAS gives conclusive evidence that chemical state of copper significantly dominates the catalytic behavior and the product profile of CO_2_RR. Our results can offer understandings of the fundamental chemical state and insights into the establishment of selective eCO_2_RR.

## Results

### Catalyst synthesis and characterization

A two-stage wet chemical synthesis was employed to realize our model catalyst. Uniform Cu nanocubes were first synthesized with an average size of 38.0 ± 4.2 nm (Fig. 1c, and Supplementary Figs. 1 and 2). To investigate the individual role of Cu(0) and Cu(1+), 1-hexadecylamine (HDA) was utilized to mediate the oxidation rate of targeted metallic nanomaterials and to gently oxidize the as-prepared Cu nanocubes^36^, which protected the morphologies of the as-prepared nanomaterials from being crushed and further suppressed the effects caused by morphologies of materials^37^. TEM image of the oxidation modified Cu nanocubes (CuO_x_ hereafter) clearly demonstrated that the morphology of CuO_x_ stayed a cube-like after the oxidation modification (Fig. 1d). Regarding the chemical composition of the as-prepared CuO_x_, selected area electron diffraction (SAED) was characteristic of two domains, metallic Cu and oxide Cu_2_O species, in the CuO_x_ (Fig. 1e). These observations confirm the formation of our desired material made of Cu–Cu(I) ensemble. Electrochemical analyses also agreed with the electron microscopic analysis. Shown in Supplementary Fig. 3a, the LSV indicated at −0.45 V (vs RHE), the system had a strong cathodic response, suggesting the existence of oxide species in CuO_x_. To identify species of such oxides, as depicted in Supplementary Fig. 3b, the CV scanning in a cathodic direction showed that in the first cycle (green line), a noticeable reduction peak which was preceded by a slight oxidation peak could be corresponding to a reduction from Cu^+^ to Cu, suggesting the oxidized copper atoms in our catalyst were nearly cuprous^38^. In the second cycle of the CV measurement (indicated in red), the original slight oxidation peak grew clearer as shown in the inset of Supplementary Fig. 3b. This growth supported the idea that our catalysts contained Cu^+^ ions, since the following reduction phenomenon also occurred and represented the reduction of Cu^+^ to Cu.

To further confirm the chemical composition, X-ray absorption spectroscopy (XAS) of Cu K-edge for the CuO_x_ was conducted (Fig. 1f and Supplementary Fig. 4). As depicted in Fig. 1f, the normalized 1st derivative of X-ray absorption near edge structure (XANES) of CuO_x_ and references suggested that no detectable Cu(II) species existed in the as-prepared CuO_x_^39^. On the other hand, a strong peak overlapping that of Cu_2_O at ~8980 eV indicated the presence of Cu(I) species in the CuO_x_ catalyst. Notably, a slight difference between the normalized absorbance at 8981 eV of CuO_x_ and that of Cu_2_O might be attributed to a couple of reasons, such as a dilution effect from metallic Cu species and the catalysts’ nanoscale^25^. Wavelet transform was employed to clarify the coordination environment near by the Cu atom and demonstrate the atomic dispersion of Cu in the CuO_x_ sample. As shown in Fig. 1g, a strong wavelet transform signal focused at 7.4 Å^−1^ derived from Cu–O contribution can be observed, while another stronger signal focused at 8.8 Å^−1^ can be attributed to a contribution of Cu–Cu. The Fourier-transformed k^3^-weighted spectra of CuO_x_ and corresponding references reveal the characteristic peaks at 2.3 Å, which correspond to the Cu–Cu scattering, implying the existence of metallic Cu (Fig. 1h). Note that a significant Fourier-transformed peak at 1.6 Å can be referred to the scattering path of Cu–O, which implied the presence of the Cu oxide with a similar nature to that of Cu_2_O. The wavelet transform contour plot of CuO_x_ also further clarified the neighboring environment around Cu, which is consistent with all observations above and indicated the fact that the copper species in the as-prepared CuO_x_ were nearly of Cu and Cu(I).

### CO2RR performance evaluation

**Fig. 2:**
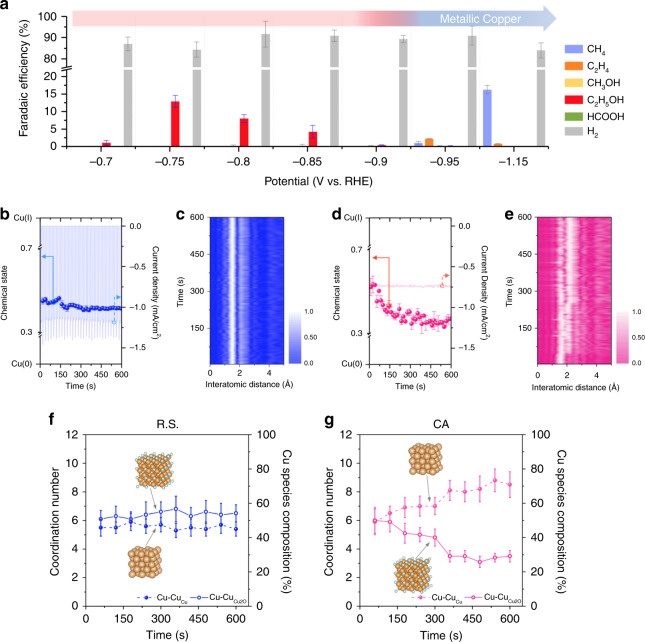
Operando time-resolved XAS characterization. **a** Potential dependence of CO_2_RR product selectivity on the CuO_x_ in 0.5 M CO_2_-saturated KHCO_3_ under the employment of redox shuttle. The error bars represented standard deviations based on three individual measurements. **b** Time-resolved variations using Redox Shuttle (R.S.) of Cu species in CuO_x_ and the corresponding electrochemical responses during CO_2_RR at −0.75 V and **c** the corresponding time-resolved EXAFS spectra without phase-correction of CuO_x_ under CO_2_RR. **d** Time-resolved variations using chronoamperometry (CA) of Cu species in CuO_x_ and the corresponding electrochemical responses during CO_2_RR at −0.75 V and **e** the corresponding time-resolved EXAFS spectra without phase-correction of CuO_x_ under CO_2_RR. The spectra were depicted in a 2D contour plot with interatomic distance (Å) on *x*-axis and with time (sec) on *y*-axis, while normalized Fourier transform magnitude served as corresponding intensity. Quantification of time-resolved chemical composition information extracted from operando Quick Cu XAS for CuO_x_ under eCO_2_RR using redox shuttle (**f**) and conventional chronoamperometry (**g**). Each error bar indicated an estimated standard deviation based on the curve fitting of EXAFS spectra. Both cathodic potentials were −0.75 V and electrolyte was 0.5 M CO_2_-saturated KHCO_3_.

To realize the steady chemical state of the as-prepared CuO_x_ and clarify the real dominated factor in CO_2_ reduction electrocatalysis, we adopted the method of switching potential (redox shuttle; R.S. hereafter). Specifically, we applied a constant anodic potential (0.5 V), which was in light of the above discussion regarding the oxidation of CuO_x_, and various cathodic potentials (from −0.7 to −1.15 V). The time interval of each anodic and cathodic potential cycle was set for 10 s (see Supplementary Fig. 6 for the potential map of R.S.). As stated above, the technique provides the electrocatalytic materials with a cycle of oxidation and reduction, thereby having the potential to stabilize the chemical nature of materials. Figure 2a exhibited the overall CO_2_RR performance of the as-prepared CuO_x_ catalyst. Surprisingly, until −0.9 V, the C_2_H_5_OH molecule was the only identified CO_2_RR product in our system while the rest product was hydrogen gas generating from proton reduction, and the corresponding F.E. (Faradaic Efficiency) reach a maximum value of ~12.9 % @ −0.75 V. With increasing the cathodic potential through the redox shuttle, the F.E. of ethanol gently declined. Note that the ethanol was still the only product from CO_2_RR. In contrast to the case of redox shuttle, the CO_2_ reduction product through a conventional chronoamperometry (CA hereafter) at the same potential of −0.75 V was carbon monoxide (Supplementary Fig. 5), which was characteristic of a copper-like behavior with the only product of CO from CO_2_RR at such potential^1,37^. The CuO_x_ catalyst in present study gave a copper-like CO_2_RR performance once applying a higher cathodic potentials (higher than −0.95 V) where the dominant CO_2_RR product was CH_4_. We could say that even though the increasing potential would bring a decline in F.E. toward ethanol, the single product from CO_2_RR still could be realized without presence of other common CO_2_RR products, such as C_2_H_4_, CO, and CH_4_. This finding further validates the fact that the redox shuttle can achieve the desired single selectivity of CO_2_RR. Note that we kept anodic potential at 0.5 V (vs RHE), referred to the oxidation potential of as-prepared CuO_x_ (as revealed in Supplementary Fig. 3), and varied the cathodic potentials from −0.7 to −1.15 V (vs RHE). Anodic and cathodic potentials were switched in an interval of 10 s (Supplementary Fig. 6). Apparently, it seems that a large cathodic potential was able to result in significant increasing the current density of CO_2_RR. Nevertheless, the large cathodic potential was failed to achieve a highly selective CO_2_RR production since that was going to cause a remarkably generation of metallic Cu^0^ (vide infra). As indicated by the CV results (Supplementary Fig. 7 and Supplementary Note 1), the oxidation variations of Cu species between applied cathodic potentials were relatively subtle through using R.S. and therefore highly suggested the technique enabled the composition of Cu species in the CuO_x_ to be well preserved. Interestingly, it is well known that the formation of C_2_H_4_ and C_2_H_5_OH may share the same path way of CO_2_RR through a hydroxyl containing intermediates^40–42^. As a result, more detail analyses in following sections are essential to address why C_2_H_5_OH is the favorable product rather than C_2_H_4_ in our system.

### Operando XAS characterization

To assess the chemical composition of the CuO_x_ under CO_2_RR, operando XAS was performed, due to its capability to probe the chemical composition of the catalysts under realistic reaction conditions^26,43–45^. As we mentioned above, conventional XAS only offers the information which is referred to a steady condition because of time requirement in spectrum acquirement (typically several tens mins). Thus, it inaccessibly provides a convincing time-resolved information for an electrochemical system where the reactions are typically being steady on a timescale of seconds or few minutes^46^. With the aim of addressing this timescale mismatch, the operando time-resolved X-ray absorption spectroscopy (operando TR-XAS) with a sub-second time-resolution can be reached by equipping a continuously oscillating crystal on a monochromator^47^. Operando time-resolved-XANES spectra for CuO_x_ during CO_2_RR at −0.75 V (vs RHE) using R.S. and those of using CA were displayed in Supplementary Figs. 8 and 9, respectively. A linear model developed by Vitova et al.^48^ was utilized to extract the pristine and time-resolved compositional information from acquired XANES spectra. As shown in Supplementary Fig. 10, the result suggested that the composition of the pristine CuO_x_ was of equal Cu(I) and Cu. This not only supported again our previous characterization that Cu(I) and Cu coexisted in the CuO_x_ (Fig. 1c–h), but also proved the feasibility of the linear model to quantitatively analyze the composition of Cu species, as Vitova et al. suggested. In this regard, we used the linear model to extract the chemical nature information of CuO_x_ from the operando time-resolved-XANES spectra. Extracted time-resolved chemical composition information combined with the electrochemical responses was shown in Fig. 2 (2b for R.S. and 2d for CA) and in Supplementary Fig. 11 (the magnified portion of Fig. 2b by which one can track the footprint of XAS acquisition under the R.S. working condition). During the CO_2_RR, R.S. restricted the chemical composition of CuO_x_ in around a composition of half Cu and half Cu(I), while the CA transformed the chemical composition of CuO_x_ into that Cu-dominated. Following the 1-hour reaction time, XANES spectrum of the CuO_x_ after the CO_2_RR using R.S. remained nearly unchanged, further indicative of R.S. having the ability to preserve the chemical composition. On the other hand, that of the CuO_x_ after the electrolysis using CA clearly showed an electrochemical reduction toward metallic Cu (Supplementary Fig. 10).

As shown in Fig. 2c, e, operando time-resolved EXAFS verified that the intensity of bond pairs regarding Cu–O at 1.6 Å and Cu–Cu at 2.3 Å stayed highly stable under the employment of R.S., whereas the intensity under the case of using CA showed a decline at 1.6 Å and an increase at 2.3 Å that could be corresponded to a transformation from Cu_2_O to metallic Cu^0^. To further quantify such chemical composition information from operando EXAFS, we obtained the Cu–Cu path from both Cu_2_O (Cu–Cu_(Cu2O)_) and metallic Cu (Cu–Cu_(Cu)_) extracted by a standard fitting procedure (detailed results shown in Tables 1–2 and Supplementary Figs. 12–31). We note here that in order to have quality spectra for EXAFAS fitting, the related XAS acquisition time was extended from 5 to 60 s. While the time resolution could not match that of seconds (Fig. 2b–e), the minutes-resolved EXAFS fitting results were also in line with those seconds-resolved (see below). The quantification was displayed in terms of coordination numbers and, more straightforward, in terms of the percentage of both Cu(0) and Cu(I) according to Eqs. (1) and (2):

$$\frac{{CN_{({\mathrm{experimental}}\,{\mathrm{Cu}} - {\mathrm{Cu}}@2.5{\AA})}}}{{CN_{({\mathrm{theorectical}}\,{\mathrm{metallic}}\,{\mathrm{Cu}})}}} \times 100\% = Cu(0){\mathrm{composition}}(\% )$$

$$\frac{{CN_{({\mathrm{experimental}}\,{\mathrm{Cu}} - {\mathrm{Cu}}@3.0{\AA})}}}{{CN_{({\mathrm{theorectical}}\,{\mathrm{Cu}}2{\mathrm{o}})}}} \times 100\% = Cu(I){\mathrm{composition}}(\% )$$

where CN_(experimental Cu-Cu@2.5 Å)_ and CN_(experimental Cu-Cu@3.0Å)_ represent the coordination numbers (CNs) of the Cu–Cu path at 2.5 Å and 3.0 Å, respectively. CN_(theoretical metallic Cu)_ and CN_(theoretical Cu2O)_ are equal to those of metallic Cu and Cu_2_O, namely 12. As depicted in Fig. 2f, g, time-resolved fitting results fairly agreed with both analyses of XANES and EXAFS. Using R.S., the CNs of Cu(I) and Cu(0) in the as-prepared CuO_x_ stayed relatively steady during CO_2_RR (Fig. 2f). By contrast, under the employment of CA, the CNs of Cu(I) and Cu(0) in the CuO_x_ gradually reduced and increased, respectively, suggesting the electrochemical reduction of the CuO_x_ (Fig. 2g). Consequently, the results of time-resolved operando XAS proved that under CO_2_RR, the chemical composition of CuO_x_ could remain in a steady state of half-and-half Cu and Cu(I) through using R.S.

**Table 1:** Operando EXAFS fitting parameter for the CuO_x_ at −0.75 V under CO_2_RR using redox shuttle approach.

Sample	Cu–Cu_Cu2O_		Cu–Cu_Cu_	
Time (s)	C.N.	*R* (Å)	C.N.	*R* (Å)
60	6.1 (6)	3.02 (2)	5.5 (6)	2.45 (3)
120	6.3 (7)	3.03 (3)	5.5 (4)	2.43 (2)
180	6.1 (5)	3.03 (2)	5.9 (6)	2.45 (3)
240	6.4 (9)	3.02 (3)	5.6 (5)	2.45 (3)
300	6.6 (7)	3.00 (3)	5.7 (5)	2.45 (2)
360	6.8 (9)	3.04 (3)	5.3 (5)	2.44 (2)
420	6.3 (6)	3.00 (3)	5.5 (6)	2.45 (3)
480	6.6 (8)	3.02 (1)	5.4 (5)	2.45 (1)
540	6.4 (8)	3.02 (3)	5.7 (6)	2.45 (3)
600	6.5 (6)	3.02 (3)	5.4 (5)	2.45 (2)

Uncertainties in the last digit are given in parentheses.

**Table 2:** Operando EXAFS fitting parameters for the CuO_x_ at −0.75 V under CO_2_RR using conventional chronoamperometry.

	Cu–Cu_Cu2O_		Cu–Cu_Cu_	
Time (s)	C.N.	*R* (Å)	C.N.	*R* (Å)
60	6.9 (9)	2.88 (4)	5.9 (9)	2.43 (4)
120	5.9 (7)	2.98 (3)	6.5 (5)	2.43 (2)
180	5.1 (7)	2.92 (3)	6.9 (7)	2.47 (3)
240	5.0 (5)	2.95 (2)	7.0 (7)	2.49 (2)
300	4.8 (6)	2.91 (3)	7.0 (6)	2.41 (2)
360	3.5 (4)	2.90 (2)	8.1 (7)	2.42 (2)
420	3.5 (4)	2.93 (3)	8.0 (7)	2.45 (2)
480	3.1 (4)	2.91 (3)	8.2 (9)	2.44 (3)
540	3.4 (3)	2.96 (2)	8.8 (8)	2.46 (2)
600	3.5 (4)	2.89 (3)	8.5 (9)	2.46 (3)

Uncertainties in the last digit are given in parentheses.

**Fig. 3:**
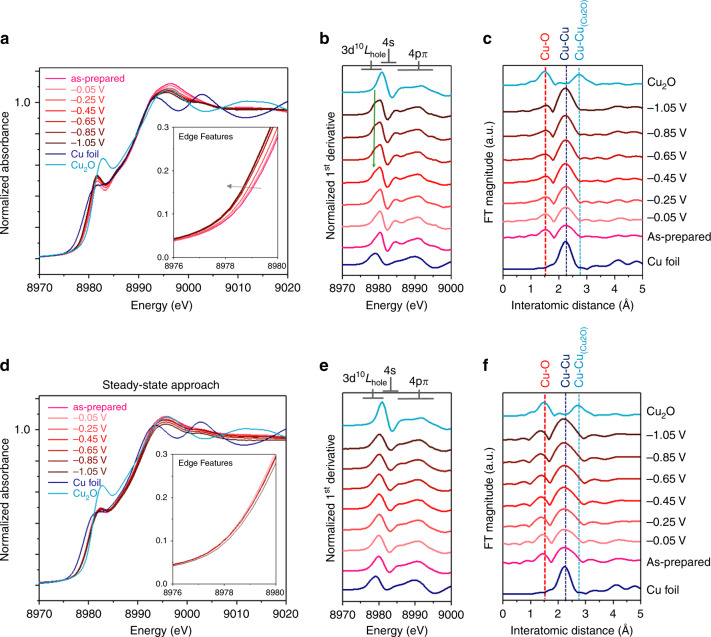
Conventional operando XAS characterization. **a** Potential dependence of operando Cu K edge XANES spectra without phase-correction of the CuO_x_ under CO_2_RR in 0.5 M CO_2_-saturated KHCO_3_ using chronoamperometry (CA) and the corresponding 1st derivative spectra (**b**). **c** Potential dependence of operando EXAFS spectra of the CuO_x_ under eCO_2_RR in 0.5 M CO_2_-saturated KHCO_3_ using CA. **d** Potential dependence of operando Cu K edge XANES spectra without phase-correction of the CuO_x_ under CO_2_RR in 0.5 M CO_2_-saturated KHCO_3_ using R.S. and the corresponding 1st derivative spectra (**e**). **f** Potential dependence of operando EXAFS spectra of the CuO_x_ under eCO_2_RR in 0.5 M CO_2_-saturated KHCO_3_ using R.S.

By contrast, under the employment of CA, the chemical composition of CuO_x_ continued a downward trend toward Cu(0). A similar result as illustrated in Fig. 3 was also revealed by the conventional XAS, showing a significant reduction of Cu^+^ to generate metallic Cu^0^. XANES data elucidated as a cathodic potential was applied from −0.05 to −1.25 V (Fig. 3a, b). The XANES features as illustrated in Fig. 3b were denoted as three distinct peak-like structures, while no remarkable pre-edge feature that caused by a dipole transition from *1s* to the mixing states of *4p* and *3d* could be visible and suggested a centro-symmetry nature of copper^37,49^. An additional small feature of the excitation in the 3d^10^L_hole_ state at a lower energy region (as indicated by green arrow) gradually intensifies with increasing cathodic voltage, this indicates the formation of metallic Cu (Fig. 3b). Furthermore, the shifts in the edge position could conclude the chemical states in CuO_x_ were reduced into copper-like and the content of metallic Cu(0) species in the CuO_x_ evidently increased. Consistent with XANES spectra, the analysis of extended X-ray absorption fine structure (EXAFS) revealed that as an applied cathodic potential increased, the metallic Cu–Cu bond pair at 2.3 Å, matching a standard metallic Cu, intensified significantly (Fig. 3c). Worth noting is that like cases in literature^39^, the as-prepared CuO_x_ exhibited a resistance to electrochemical reduction, as the intensity of a Cu–O bond pair at ~1.6 Å, referring to a Cu_2_O standard, did not entirely disappear even under a cathodic environment. Despite having this resistance to the electrochemical reduction, however, the as-prepared CuO_x_ could not remain its initial chemical nature under CO_2_RR when the CA was adopted. This observation can refer to the fact that the catalytic sites and the chemical states can fail to reach a steady situation under the conventional chronoamperometry. These results also can explicate the finding from numerous reports that the products profile of CO_2_RR are commonly characteristic of a mixture nature.

To extend the investigation further, we also performed conventional operando XAS analyses for the CuO_x_ under CO_2_RR using R.S. in various potentials (Fig. 3d–f). Only slight variations on XAS results across the various applied potentials, the chemical composition of CuO_x_ could remain in a steady condition of the composition of Cu and Cu(I) through using R.S. as illustrated in Fig. 3d, e. Note that, as compared with those of Fig. 3b, no additional feature of the excitation in the 3d^10^L_hole_ state at a lower energy region can be found with increasing cathodic voltage, this further indicates the chemical nature of CuO_x_ can reach steady-state under CO_2_RR using R.S. (Fig. 3e). In the case of operando EXAFS spectra, the final condition from various applied potentials were similar except for the situations in high cathodic potentials (<−0.95 V vs RHE). This finding corroborated the observation of potential-dependent product profiles in Fig. 2a, in which the CO_2_RR products have become a metallic copper-like profile with a mixed feature. Therefore, these results became solid evidence to conclude the existence of the steady composition of Cu species under CO_2_RR using R.S. Aside from spectroscopic analysis, the post electron microscopy analysis also supported the validation of R.S. As shown in Supplementary Fig. 32, the TEM image of CuO_x_ after a 6-h CO_2_RR electrolysis at −0.75 V using R.S. demonstrated that the morphology of the CuO_x_ was similar to those before the electrolysis (Fig. 1d). This consistence fairly helped rule out the possibility that the observed CO_2_RR performance in the present study was brought by morphology change^37^. Meanwhile, the HR-TEM analysis, as well as related SAED, of the post-catalysts in Supplementary Fig. 33 also confirmed the chemical nature of the CuO_x_ was the same as the as-prepared catalyst (Fig. 1e). Furthermore, in terms of electrochemical stability, we also evaluated the electrochemical active surface area (ECSA) of CuO_x_ before and after the CO_2_RR electrolysis. The double layer capacitance (*C*_dl_) was used for the assessment, since it is directly proportional to the ECSA of materials^50,51^. Using a cyclic voltammetry (CV) method, the *C*_dl_ values of CuO_x_ before and after the electrolysis were 77.27 and 76.60 μF cm^−2^, respectively (Supplementary Figs. 34 and 35). The result of nearly the same *C*_dl_ values reflected the fairly stable ECSA of CuO_x_ after the electrolysis and highly suggested the stability of using R.S. Altogether, results of the operando XAS analyses, the electron microscopy analyses, and electrochemical studies convincingly proved the validation of R.S. as a steady-state approach.

Through operando TR-XAS analyses, we correlated the chemical nature of CuO_x_ with its unique selectivity toward asymmetric C_2_H_5_OH molecules, which has evidently concluded a paramount role played by the composition of the Cu–Cu(I) ensemble. Moreover, the preceding analyses also revealed that different CO_2_RR products may result from catalytic surfaces with different compositions of Cu and Cu(I); a Cu-dominated surface synthesized CO, while the surface with halves of Cu and Cu(I) gave rise to ethanol (Supplementary Fig. 10). This rather suggests a delicate Cu(I)/Cu(0) interface is the likely active center for CO_2_ to asymmetric products and motivates future work regarding such interfacial engineering. Analogous interfacial engineering strategies have been applied to enhance product selectivity in electrochemical reaction systems^52,53^. The study by Feng et al., for example, has demonstrated a linear correlation between the boundary densities within their as-prepared Cu nanoparticles and the corresponding catalytic behavior of reducing CO to multi-carbon products, i.e., ethanol and acetate^54^. Furthermore, using scanning electrochemical cell microscopy (SECCM), Mariano and coworkers provided a submicrometer-resolved study on gold films^55^. The study with such spatial resolution clearly demonstrated that the electrocatalytic activity of a gold film for CO_2_RR depends on the grain boundary types and densities on the film.

### Theoretical understanding of the CO2RR selectivity

As follows, we make an attempt to elucidate the unique selectivity in our reaction system thorough theoretical computation. By using density functional theory (DFT) calculations, we further evaluated the stability of oxygen-vacancy generation/removal with various compositions of Cu and Cu(I) at the top layer of Cu_2_O. All details regarding the structural simulation are addressed in Methods, while the model coordinates are listed in Supplementary Table 1. Such a geometric stability during the oxygen removal process can potentially facilitate the regeneration of the acting-catalytic environment by the redox-shuttle approach. These structural simulation results demonstrated that the equal numbers of Cu and Cu(I) on the top layer could provide an ideal environment to host the C–C bond formation intermediate (OCCO) (Supplementary Figs. 36 and 37). All studies in following parts, therefore, will focus on the surface composed of the equal numbers of Cu and Cu(I) for subsequent modeling (defined it as a Cu–Cu(I) ensemble). Afterwards, we characterized C-C coupling behavior on the Cu–Cu(I) ensemble, and the configurational results—calculated absorption energy and geometrically optimized structures of absorbed CO molecules at the gas-solid interface – were shown in Supplementary Fig. 37. The ensemble configured dimerized CO molecules to an asymmetric “end-side” configuration; a CO molecule was absorbed in the side-on direction and coupled with an end-on CO molecule, which was consistent with previous work^56^.

**Fig. 4:**
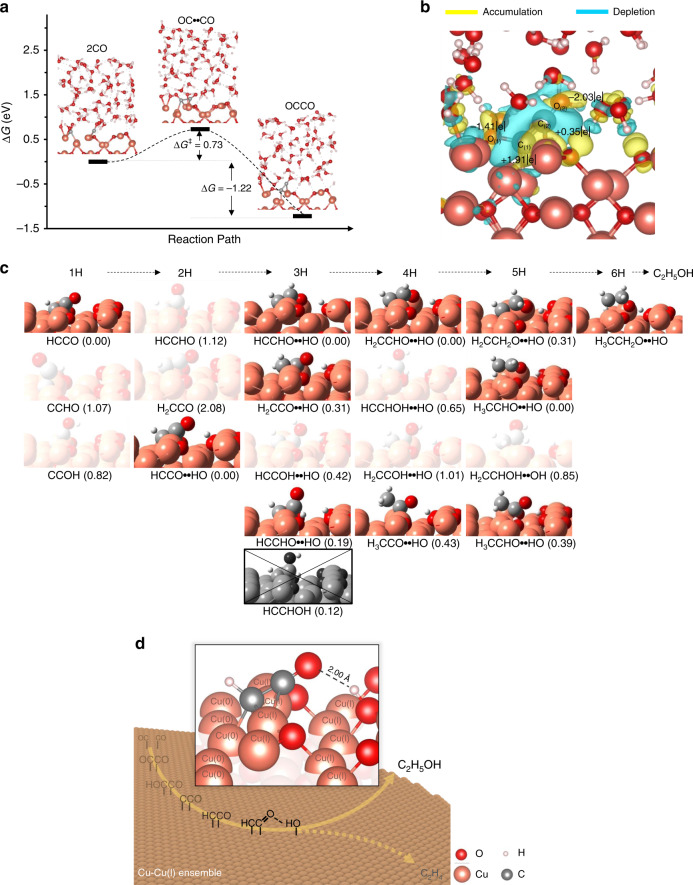
DFT calculation results and proposed selectivity mechanism scheme. **a** The profiles of the potential energy surface for the dimerization of CO on Cu–Cu(I) ensembles. **b** The Bader chargers of the OCCO intermediates. **c** The accessible carbonyl-stabilized intermediates on the Cu–Cu(I) ensemble. The relative free energy in eV of each hydrogenation stage is denoted in the parentheses. **d** The schematic representations of the determined factor for the selective ethanol synthesis.

On the other hand, the explicit water solvation effect has been demonstrated to well achieve a realistic modeling in free energy calculations. As depicted in Fig. 4a, the free energy barriers (ΔG^‡^) for the C–C bond formation under the explicit water solvation indicated the overall OCCO formation on the ensemble was thermodynamically accessible at 0.73 eV, as the corresponding reaction free energy ∆*G* was −1.22 eV (for computation details, refer to Supporting Information). Furthermore, Bader charge analysis also suggests that the mixed-valence ensemble constructively stabilize the asymmetrical OCCO intermediate though a favorable electrostatic interaction, in which the highly polar OCCO (+0.50 and −1.68 for the positive and negative fragments of (O_(1)_C_(1)_)^δ+^(C_(2)_O_(2)_)^δ−^, respectively) is stabilized by the presence of Cu(0) and Cu(I) on the surface, respectively (Fig. 4b). All computational results demonstrated the potential introduced by the mixed-valence ensemble could produce an asymmetric OCCO intermediate in CO_2_RR, which might considerably facilitate the formation of C_2_ products from CO_2_RR.

The plausible mechanistic steps of ethanol formation are computationally explored with considering the various hydrogenated intermediates on the Cu–Cu(I) ensemble, being simulated by the mixed-valence Cu_2_O surface model, as highlighted as 1H to 6H in Fig. 4c. The calculated electrochemical step started from the hydrogenation step of CCO (1H), being generated from the dehydration step of the experimental observed OCCOH intermediate^54^. The relative free energies in respect to the minimum geometries (all marked as 0 eV) at each hydrogenation stage were labeled in Fig. 4c. It should be noted that all of the hydroxyl containing intermediates (CCOH, HCCOH_OH, HCCHOH_OH, H_2_CCOH_OH, and H_2_CCHOH_OH) were found to be less stable by 0.82, 0.43, 0.65, 1.08, and 0.79 eV than the corresponding minima, respectively, where the presence of hydroxyl groups within the hydrocarbon species could lead to the pathway of ethylene formation via a proton-coupled-electron dehydration process. Although HCCOH (crossed out in grayscale) was proposed as a necessary intermediate for C_2_H_4_ formation^55^, it became inaccessible due to its unfavorable precursor – HCCHO.

By contrast, OH species at the boundary of Cu–Cu(I) ensemble could play a critical role in facilitating the ethanol formation. Such a mixed-valance region favored the formation of surface hydroxyl given the electron-rich Cu region enhances the basicity of the boundary oxygen sites of Cu(I) region. Theoretically, OH species at the Cu–Cu(I) boundary was commonly identified along the hydrogenation pathway of CO_2_ reduction, and that resulted in the attractive electrostatic interactions between surface-OH dipole and the dipole of carbonyl group of hydrocarbon intermediates. This carbonyl stabilization effect could protect the oxygen end from being protonated as well as occupy the valence electron of carbon to avoid the CC double bond formation. Consequently, managing the boundary oxygen sites during the electrochemical CO_2_ reduction could be the determined factor of ethanol generation. The unique selectivity toward C_2_H_5_OH could be attributed to the fact that the CuO_x_ could maintain well balance of Cu and Cu(I) during eCO_2_RR using R.S. and offer an asymmetrically OCCO adsorbing site with the subsequent carbonyl group stabilization effect by the OH groups at the boundary of Cu–Cu(I) ensemble, eventually synthesizing an asymmetric oxygenated product (Fig. 4d).

## Discussion

In summary, using redox shuttle manner, the results of product characterization demonstrated that in a wide potential range, the as-prepared CuO_x_ exclusively produced C_2_H_5_OH over other CO_2_RR products. Using the operando time-resolved XAS, we tracked the evolution of its chemical nature under CO_2_RR. Quantitative XANES and EXAFS analyses evidently revealed that the steady chemical composition of the material could be achieved via the usage of the redox shuttle approach, while the employment of conventional chronoamperometry drastically altered the chemical nature of the material. Collectively, a strong correlation between the stabilized ensembles and the corresponding unique selectivity of CO_2_RR products has been established. More importantly, the carbonyl stabilization mechanism by the boundary OH species was proposed to prevent the protonation of the terminal oxygen sites of hydrocarbons, and such a hedge device produced an oxygenated CO_2_RR product (ethanol in our case) with the unique selectivity. Our studies provided the first empirical real time information, correlating the chemical nature of Cu-based catalysts with their corresponding selectivity toward CO_2_RR products.

## Methods

### Chemicals

The copper(I) bromide (CuBr, 98%), copper(I) chloride (CuCl, 99%), trioctylphosphine (TOPO, 99%), 1-hexadecylamine (HAD, 90%), oleylamine (80-90%), 1, 5-pentanediol (98%), and nafion solution (20 wt. %) were purchased from Acros Organics. The potassium bicarbonate (KHCO_3_, 99%) was purchased from Fisher Scientific. The gases CO_2_ (99.999%) and N_2_ (99.999%) were purchased from Shen-Yi Gas Co. These chemicals were used without further purification.

### Synthesis for copper nanocubes

The nanocubes were prepared through a polyamine process under an inert atmosphere. CuBr (1 mmol) and TOPO (3 mmol) were firstly dissolved into oleylamine (15 mL) in a three-neck round-bottom flask. After well mixed, the solution was flushed with N_2_ for 30 mins to remove the oxygen in the apparatus. Under the inter atmosphere, the solution was heated at 80 °C for 5 mins and then up to 230 °C for another 3 h, turning into a reddish-brown clouded solution. Afterwards, the solution was air-cooled to room temperature for purification. The solution was washed with n-hexane (40 mL) and centrifuged at 8000 r.p.m. for 10 mins three times. The purified nanocubes were stored in n-hexane for the later use.

### Synthesis for mixed-valence copper nanomaterials

In all, 20 mg HDA were added into 9 mL 1, 5-pentanediol. Then, the solution was preheated at 170 °C for 10 mins. Afterwards, as-prepared copper nanocube solution (1 mL) was transferred into the solution via one-shot injection. The solution was kept at the temperature for a half hour. Eventually, the solution turned yellow, suggesting the formation of mixed-valence copper nanomaterials. The materials were washed with an ethanol-water mixture in ratio 7:3 and centrifuged at 10,000 r.p.m. for 10 min three times. The purified materials were kept in absolute alcohol for the later use.

### Catalyst characterization

The morphology of as synthesized materials was characterized using the transmission electron microscopy (TEM, Hitachi H-760 operated at 80 kV) and the scanning electron microscopy (SEM, JSM-6700F). The materials’ crystal structure was studied by using selected area electron diffraction (SAED) in a high-resolution TEM (HRTEM, JEOL JEM-2100F operated at 200 kV). The elemental analysis of the materials was conducted using energy-dispersive X-ray spectroscopy (EDS) inside the HRTEM.

### Electrochemical measurements

All electrochemical measurements were carried out using Biologic-VSP instrument and in a three-electrode setup. The working electrode was a glassy carbon electrode (0.782 cm^2^); the counter electrode, a Pt wire; the reference electrode, an Ag/AgCl electrode (in 3 M KCl). The test cell was a home-made electrochemical cell. The cell consisted of two parts, cathode and anode compartments. The working electrode and reference electrode were placed into the cathode compartment, and the counter was in the anode compartment. Both compartments were separated by an anion exchange membrane (Fumasep FAA-3-PK-130, FUMATECH) to prevent potential contamination resulting from the dissolution of Pt counter. In electrochemical experiments, 10 μL of the solution of as-prepared materials (2.5 M) were loaded on the glassy carbon. Each compartment was filled with 30 mL pre-bubbled CO_2_-saturated 0.5 M KHCO_3_, whose pH changed from 8.6 to 7.2, and the CO_2_ flow was kept at 100 sccm for 10 more mins in the cathode compartment to build an air-free atmosphere for the later detection of as-prepared materials’ electrocatalytic activity for CO_2_ reduction. Several electrochemical techniques were employed in the study, including cyclic voltammograms (CV), chronoamperometry (CA), and electrochemical redox shuttle (R.S.). CVs were conducted in the potential interval between −0.35 V and −1.05 V at a scan rate 100 mV/s. CA was performed at stated potentials. R.S. was adopted at stated potentials and at stated time intervals (one may refer to Supplementary Fig. 6 for the potential map). Potentials listed in the article were iR-corrected and referenced to the RHE (short for reversible hydrogen electrode) scale by Eq. (3). The pH of 7.2 was used for the conversion, since the value before and after CO_2_RR treatment stayed constant at 7.2. The iR drop was determined using impedance measurement technique (ZIR) available in the potentiostat.

$$E_{{\mathrm{RHE}}} = E_{{\mathrm{Ag}}/{\mathrm{AgCl}}} + 0.21 + 0.592 \times {\mathrm{pH}} - iR$$

#### Double layer capacitance (*C*_dl_) Measurement

A cyclic voltammetry (CV) method was used to study the *C*_dl_ of as-prepared catalysts. CVs were conducted under N_2_-saturated 0.5 M KHCO_3_ at various scan rates (10–300 mV/s). A potential window from 0.1 to 0.2 V was adopted due to the absence of redox peaks in this region (Supplementary Fig. 3). Later, the anodic and cathodic current density difference of each CV at the center of the potential window was plotted as a function of scan rate, whose slope will automatically give the C_dl_.

### CO2RR product analysis

Analytical methods for product characterization here have been outlined previously^37^. Gaseous products were evaluated using gas chromatography (GC, Agilent 7890B). The GC was equipped with a thermal conductivity detector (TCD) and a flame ionization detector (FID) in series. The TCD was for H_2_ and CO detection, and the FID was for CH_4_ and C_2_H_4_ detection. As for those products in liquid phase, gas chromatography-mass spectrometry (GC-MS, 5977A) was adopted to detect ethanol and propanol, and nuclear magnetic resonance (NMR, Bruker Advance III 500 MHz) was used for the detection of formic acid. The faradaic efficiency (F.E.) of each product was calculated by Eq. (4):

$${\mathrm{FE}}(\% ) = \frac{{{\mathrm{moles}}\,{\mathrm{of}}\,{\mathrm{target}}\,{\mathrm{products}} \times n \times F}}{C} \times 100\%$$

where *n* is the number of electrons transferred, *F* stands for Faraday’s constant (96485 C mol^−1^), and *C* represents the total amount of charge passed through a working electrode.

### Operando X-ray absorption spectroscopy

Operando X-ray absorption experiments for Cu K-edge measurement were conducted at beamline BL 17C of National Synchrotron Radiation Research Center (NSRRC). A three-electrode arrangement (the same setup as those described in electrochemical measurements) was used for the operando measurements; the measurements were performed in customized Teflon reactors with a Kapton tape window, allowing X-ray transmission.

### Operando quick-scanning X-ray absorption spectroscopy

The measurements for Cu K-edge absorption were taken at TPS 44A, Hsinchu, Taiwan. The corresponding data were recorded in total-fluorescence-yield mode. The setup of the experiments was stated in the article. To have a quality spectrum, we applied the Quick-XAS mode in a time-resolution of 5 s for XANES and EXAFS analyses, while for EXAFS fitting the time-resolution was chosen to be 60 s. The X-ray absorption experimental data were collected in total-fluorescence-yield mode, in which the metallic Cu foil was taken as reference to calibrate the energy scale. Using Demeter, all X-ray absorption spectra were processed by subtracting the baseline of pre-edge and normalizing that of post-edge; such data processing was for both X-ray absorption near edge spectra (XANES) and extended X-ray absorption fine structure (EXAFS) analyses. EXAFS analysis was carried out using Fourier transform on *k*^*3*^-weighted EXAFS oscillations to assess the contribution of each bond pair to Fourier transform peak. The curve fitting of EXAFS spectra was conducted using the software, REX2000, with FEFF8 program.

### Theoretical analysis

All calculations were performed with the DFT plane-wave method utilizing the Vienna ab-initio simulation package (VASP)^57–60^. We used the projector-augmented-wave method (PAW)^61,62^ in conjunction with generalized gradient-approximation (GGA) calculation, and Perdew-Burke-Ernzerhof (PBE) exchange-correlation functional was applied. For the design of slab models, we used 8, 6, and 12 atomic layers for Cu_2_O(111), Cu_2_O(110), and Cu_2_O(100), respectively, as shown in Supplementary Fig. 36. For the DFT + U calculations, we chose the cutoff energy at 500 eV with 8 × 8 × 8 and 4 × 4 × 1 k-point mesh for bulk and surfaces models, respectively. On-site Columbic correction (*U* = 5.2 eV) was used to prevent delocalization of Cu d-orbitals, that has been demonstrated for modeling the previous Cu_2_O system^63,25^. The bottom two atomic layers were kept frozen with the remaining layers fully relaxed for these Cu_2_O slabs. In all cases, the slab calculations included a vacuum region of thickness greater than 15 Å, large enough to ensure no interaction between the two slabs. The adsorption energies were calculated based on the following equation,

$$\Delta E{\mathrm{ads}} = E[{\mathrm{slab}} + {\mathrm{adsorbate}}] - (E[{\mathrm{slab}}] + E[{\mathrm{adsorbate}}])$$

in which *E*[slab + adsorbate], *E*[slab], and *E*[adsorbate] were the calculated electronic energies of adsorbed species on the above surfaces, clean surfaces, and free molecules, respectively. The climbing-image-nudged-elastic-band (CI-NEB) method^25,64–66^ was applied to locate transition structures; the profile of the potential-energy surface (PES) was constructed accordingly. Frequency calculations were applied to verify the adsorbed intermediates and the transition states (with only one imaginary frequency). Based on the stepwise oxygen-vacancy formation energy, the Cu_2_O(110) surfaces was found to contain the most subtle surface reconstruction among the three common Cu_2_O cases (i.e., (111), (110), and (100)) as illustrated in Supplementary Fig. 36.

### Solvent model

To provide an appropriate solvent effect of CO dimerization reaction, we constructed an explicit water layers (64 water molecules) to represent the hydrogen bond network of the aqueous environment. Water molecules were described by SPC-3f(α) models^67^ using LAMMPS molecular dynamics simulator^25^. The liquid-solid interfaces were brought to equilibrium with 2 ns pre-equilibrium simulations^68^ at 350 K while the solid geometry (with adsorbate) remained frozen during the molecular dynamics simulations, being represented by atomic charges (from Bader charges of DFT). The adsorbate and solid structures were also taken from our DFT calculations. After 2 ns of pre-equilibrium, we sampled 10 solvation structures (0.2 ns apart per sample) for the initial state 2CO (IS), C–C bond coupling transition state (TS), and OCCO final state (FS), followed by solvent structure optimization at DFT-level. Finally, we took the average energy of these 10 structures to represent E(IS), E(TS), and E(FS) for CO‒CO coupling process, respectively.

### Thermodynamic correction

The relative Gibbs energy in solution (Δ*G*) for the free energy difference between any adsorbate pair on the surfaces was defined as Δ*G* = Δ*H* ‒ *T*Δ*S*. Δ*H* was estimated as Δ(*E* + ZPE + *E*_vib_) with *E* representing the average electronic energy of DFT under explicit solvation treatment, ZPE representing zero-point energy, and *E*_vib_ representing the internal energy correction of vibrational models (the relative translational and rotational contribution for any adsorbate pair was assumed canceled out)^69,70,25^. Δ*S* was predicted by the vibrational partition function using harmonic vibrations. For the CO‒CO coupling process, the vibrational modes of two surface Cu atoms were taken into account. For example, $\overline {\Delta G^\ddagger } = \overline {\Delta G} ({\mathrm{TS}}) - \overline {\Delta G} (2{\mathrm{CO}}) = [\overline {\Delta H} ({\mathrm{TS}}) - T\overline {\Delta S} ({\mathrm{TS}})] - [\overline {\Delta H} (2{\mathrm{CO}}) - T\overline {\Delta S} (2{\mathrm{CO}})]$ was estimated by each 10 sampled configurations. For the ethanol formation pathway, we did not take into account the explicit solvation effect due to its trivial difference.

## Supplementary information

Supplementary Information

## Data Availability

The data that support the findings of this study are available from the corresponding author upon reasonable request.
