# Supplementary material for: Operando time-resolved X-ray absorption spectroscopy reveals the chemical nature enabling highly selective CO2 reduction
Source: Nat Commun. 2020 Jul 14;11:3525. doi: 10.1038/s41467-020-17231-3 (PMC7360608; doi:10.1038/s41467-020-17231-3)
Supplement: Supplementary file 1 — Supplementary Information [file 41467_2020_17231_MOESM1_ESM.pdf]

Supplementary Information

Operando time-resolved X-ray absorption spectroscopy reveals the chemical nature enabling highly selective CO_2 reduction

Lin *et al*.

Table of Contents

Supplementary Tables 3

Supplementary Figures 5

Supplementary Note 1 30

Supplementary Tables

Supplementary Table 1. Cartesian coordinate of Cu/Cu(I) ensemble.

ELEMENT	*X*	*Y*	*Z*	ELEMENT	*X*	*Y*	*Z*
**Cu**	3.232	2.999	0.000	**Cu**	11.931	5.992	6.168
**Cu**	3.232	6.019	0.000	**Cu**	7.175	2.871	6.035
**Cu**	3.232	9.038	0.000	**Cu**	12.090	3.035	6.353
**Cu**	3.232	12.057	0.000	**Cu**	7.154	9.092	6.023
**Cu**	7.502	2.999	0.000	**Cu**	12.187	8.870	6.356
**Cu**	11.772	2.999	0.000	**Cu**	7.818	11.988	6.002
**Cu**	7.502	6.019	0.000	**Cu**	11.984	0.026	6.137
**Cu**	11.772	6.019	0.000	**Cu**	1.085	10.800	7.105
**Cu**	7.502	9.038	0.000	**Cu**	1.342	7.445	7.190
**Cu**	11.772	9.038	0.000	**Cu**	1.531	4.208	7.262
**Cu**	7.502	12.057	0.000	**Cu**	1.151	1.419	7.085
**Cu**	11.772	12.057	0.000	**Cu**	4.736	10.735	7.166
**Cu**	5.367	10.548	1.510	**Cu**	10.237	10.492	7.430
**Cu**	9.637	10.548	1.510	**Cu**	4.746	7.694	7.360
**Cu**	5.367	7.528	1.510	**Cu**	10.219	7.436	7.495
**Cu**	9.637	7.528	1.510	**Cu**	4.763	4.263	7.378
**Cu**	5.367	4.509	1.510	**Cu**	10.164	4.516	7.482
**Cu**	9.637	4.509	1.510	**Cu**	4.761	1.241	7.176
**Cu**	5.367	1.490	1.510	**Cu**	10.176	1.403	7.437
**Cu**	9.637	1.490	1.510	**O**	2.165	2.999	1.510
**Cu**	1.097	10.548	1.510	**O**	0.030	6.019	1.510
**Cu**	1.097	7.528	1.510	**O**	2.165	9.038	1.510
**Cu**	1.097	4.509	1.510	**O**	0.030	12.057	1.510
**Cu**	1.097	1.490	1.510	**O**	6.435	2.999	1.510
**Cu**	3.268	6.009	3.066	**O**	10.705	2.999	1.510
**Cu**	3.220	3.006	3.027	**O**	4.300	6.019	1.510
**Cu**	3.197	8.990	3.045	**O**	8.570	6.019	1.510
**Cu**	3.195	12.059	2.980	**O**	6.435	9.038	1.510
**Cu**	7.579	6.014	3.122	**O**	10.705	9.038	1.510
**Cu**	11.783	6.008	3.038	**O**	4.300	12.057	1.510

**Cu**	7.457	2.983	3.109	**O**	8.570	12.057	1.510
**Cu**	11.650	3.040	3.116	**O**	0.004	8.930	4.577
**Cu**	7.458	9.022	3.109	**O**	2.024	12.053	4.375
**Cu**	11.664	8.954	3.116	**O**	12.773	3.064	4.589
**Cu**	7.538	12.028	3.100	**O**	2.248	5.985	4.616
**Cu**	11.778	12.047	3.033	**O**	4.192	8.968	4.577
**Cu**	5.305	10.504	4.619	**O**	8.500	9.007	4.669
**Cu**	9.611	10.527	4.586	**O**	6.469	11.992	4.636
**Cu**	5.394	7.514	4.628	**O**	10.752	12.022	4.546
**Cu**	9.610	7.485	4.600	**O**	4.275	2.983	4.517
**Cu**	5.332	1.420	4.622	**O**	8.499	2.959	4.668
**Cu**	9.605	1.436	4.596	**O**	6.525	6.003	4.673
**Cu**	5.434	4.464	4.615	**O**	10.750	5.985	4.547
**Cu**	9.608	4.486	4.608	**O**	5.854	2.773	7.315
**Cu**	0.980	10.524	4.515	**O**	11.065	2.962	7.850
**Cu**	1.117	7.450	4.683	**O**	9.106	5.998	7.384
**Cu**	0.966	1.488	4.521	**O**	5.817	9.196	7.294
**Cu**	1.104	4.526	4.717	**O**	11.175	8.963	7.858
**Cu**	3.001	2.649	6.015	**O**	9.158	11.965	7.287
**Cu**	2.891	9.388	6.055	***a***	12.810	0.000	0.000
**Cu**	3.019	12.054	6.104	***b***	0.000	12.077	0.000
**Cu**	7.860	6.004	6.029	***c***	0.000	0.000	22.548

Supplementary Figures

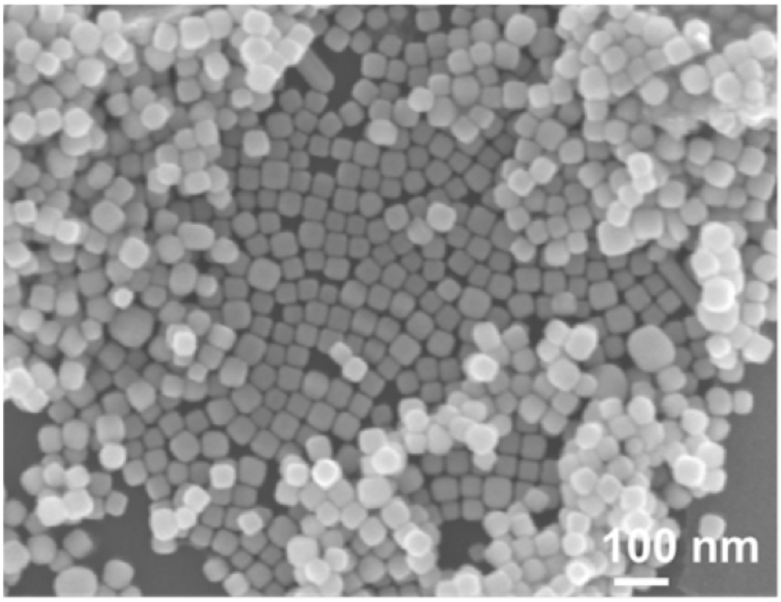

Supplementary Figure 1. SEM image of Cu nanocubes.

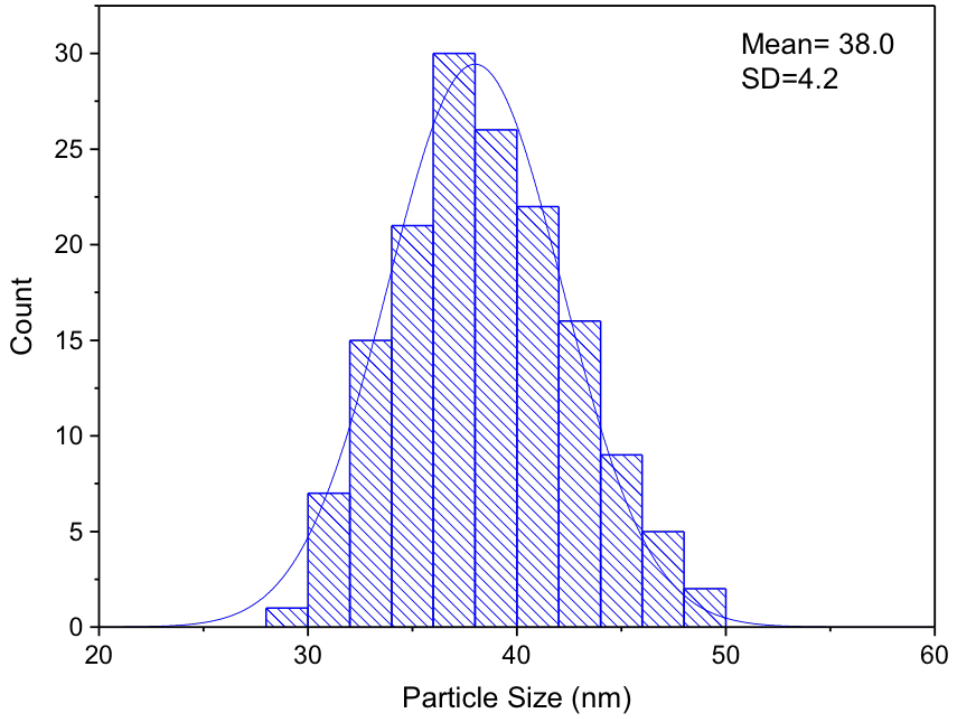

Supplementary Figure 2. Particle size distribution statistics of Cu nanocubes.

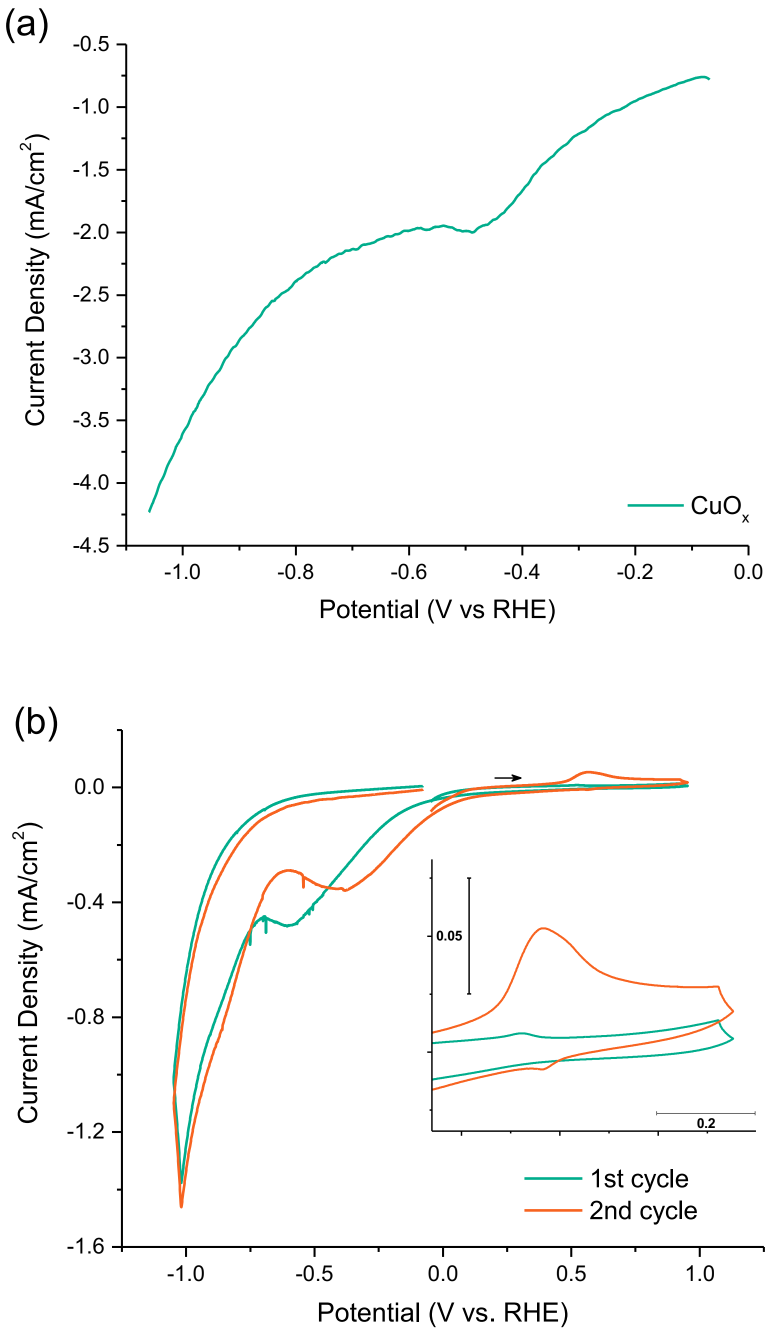

Supplementary Figure 3. (a) Linear sweep voltammetry (LSV) of the CuO$_x$. Scan rate: 100 mV/s. Solution composition: 0.5 M CO_2-saturated $KHCO_3$. (b) Cyclic voltammograms (CV) of the CuO$_x$ recorded in 0.5 M CO_2-saturated $KHCO_3$ at 100 mV/s. In the inset, zoom of the current density values.

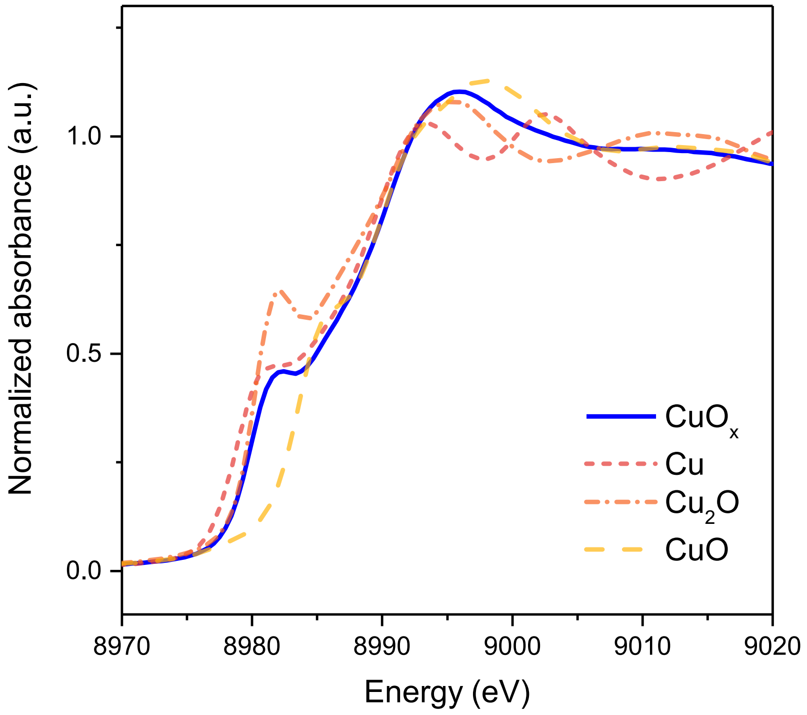

Supplementary Figure 4. The Cu K-edge XANES spectra of CuO_x and of Cu foil, commercial Cu_2O and CuO as references.

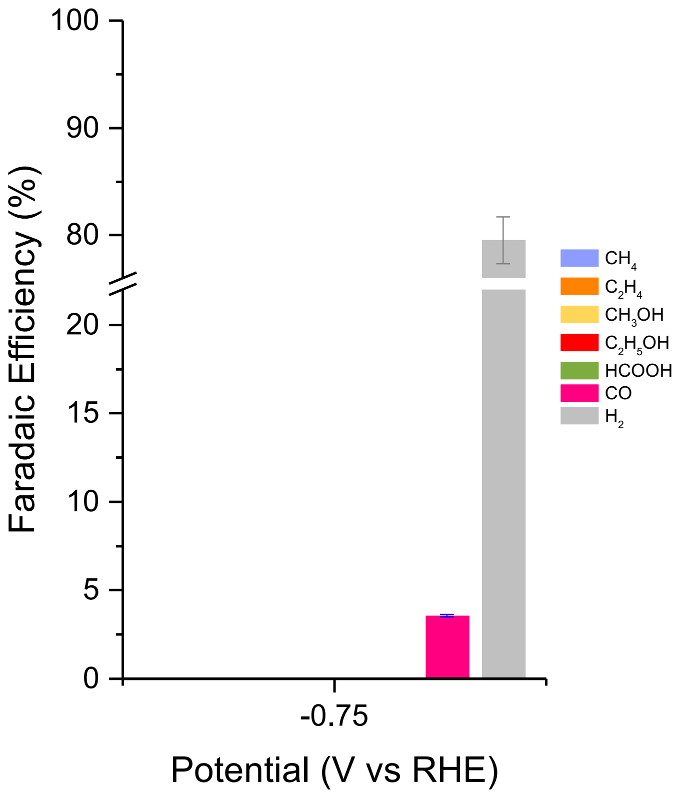

Supplementary Figure 5. eCO_2RR performance of CuO_x at -0.75 V in 0.5 M CO_2-saturated $KHCO_3$ using chronoamperometry. The error bars represented standard deviations based on three individual measurements.

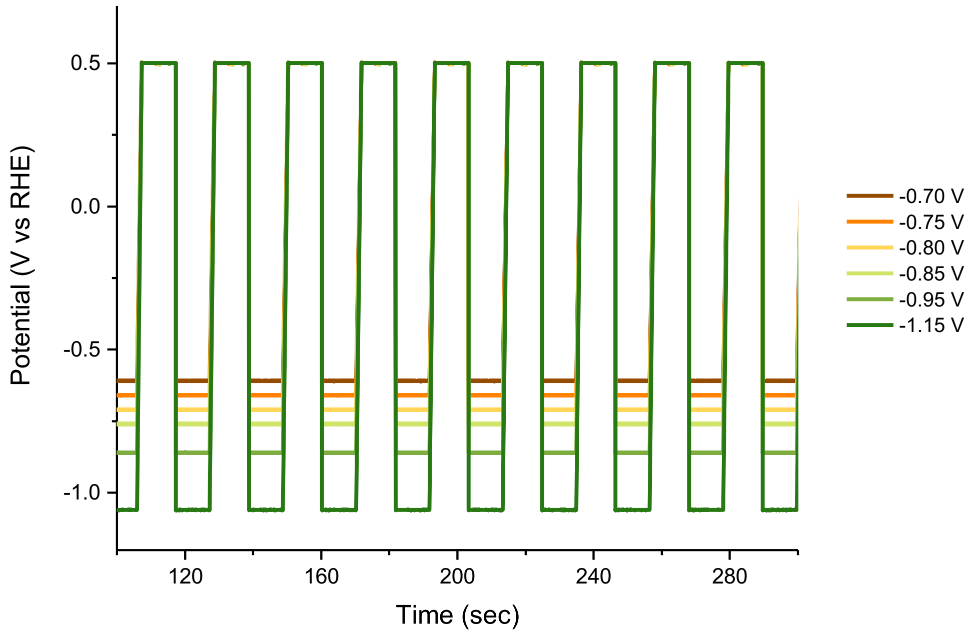

Supplementary Figure 6. Potential map of redox shuttle approach with a constant anodic potential (0.5 V vs RHE) and various cathodic potentials as indicated in the figure. The time period of each potential set was 10 seconds.

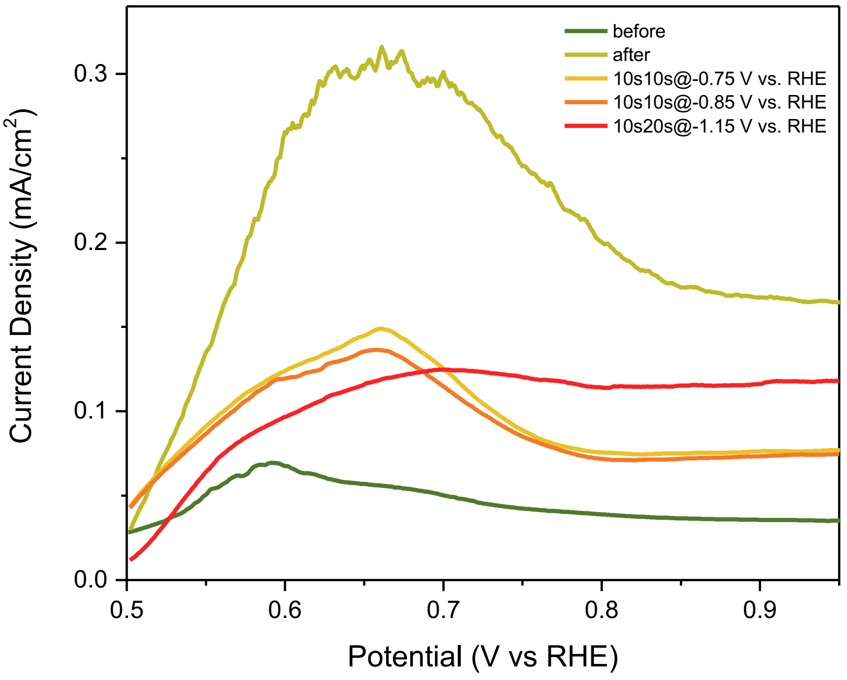

Supplementary Figure 7. Cyclic voltammograms (CV) of CuO_x recorded in 0.5 M CO_2-saturated $KHCO_3$ at 100 mV/s using various techniques.

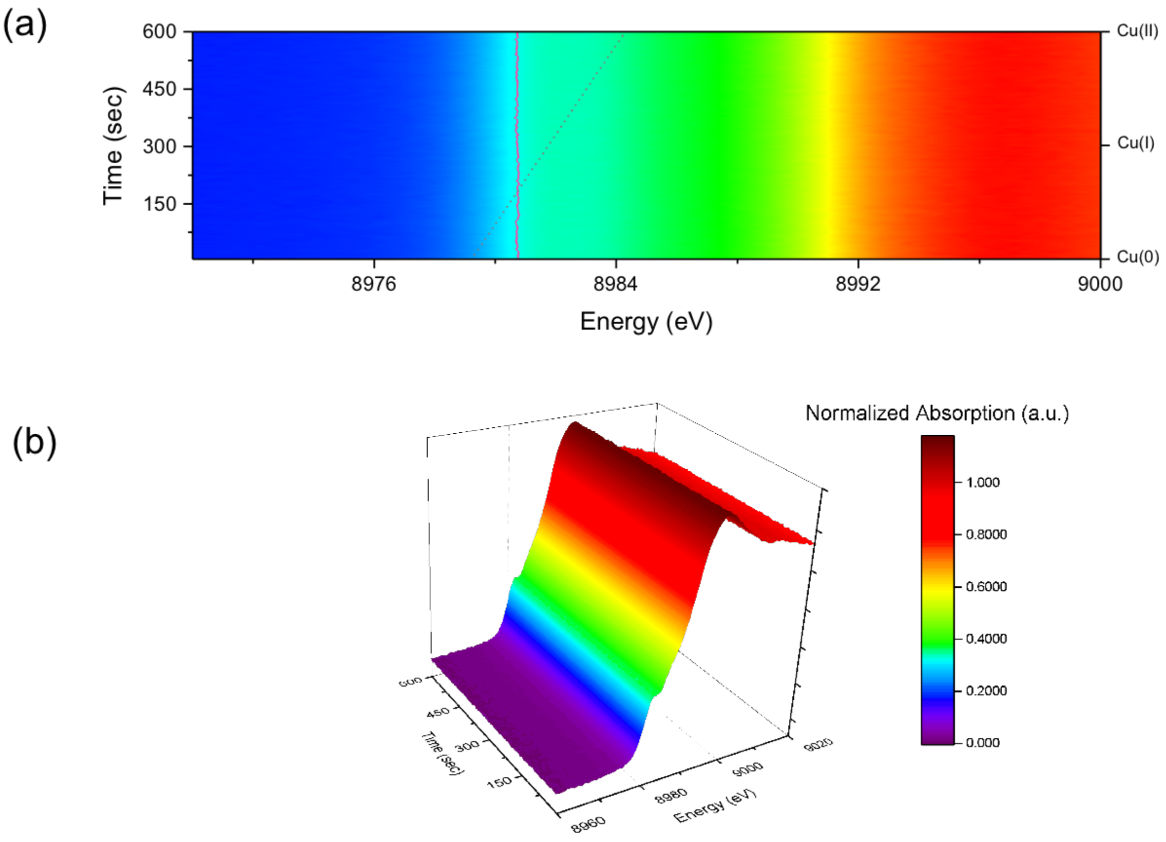

Supplementary Figure 8. (a) The double-y plot of the 2D contour plot of operando quick-scanning Cu K-edge XANES spectra of CuO_x under eCO_2RR in 0.5 M CO_2-saturated $KHCO_3$ at -0.75 V using Redox Shuttle (R.S.). The y-axis in the left represents time, while the y-axis in the right represents Cu, Cu_2O and CuO references. (b) The 3D surface plot of *operando* quick-scanning Cu K-edge XANES spectra of CuO_x under eCO_2RR in 0.5 M CO_2-saturated $KHCO_3$ at -0.75 V using R.S..

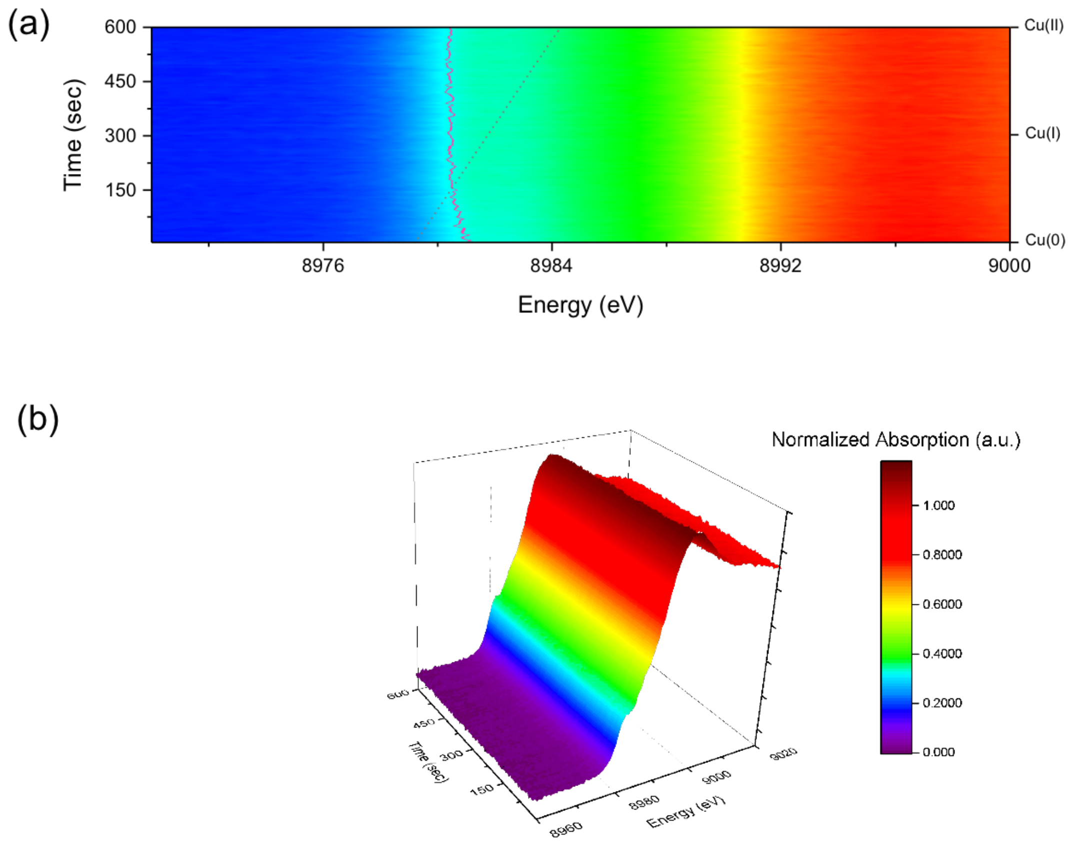

Supplementary Figure 9. (a) The double-y plot of the 2D contour plot of operando quick-scanning Cu K-edge XANES spectra of CuO_x under eCO2RR in 0.5 M CO_2-saturated $KHCO_3$ at -0.75 V using conventional chronoamperometry (CA). The y-axis in the left represents time, while the y- axis in the right represents Cu, Cu_2O and CuO references. (b) The 3D surface plot of *operando* quick-scanning Cu K-edge XANES spectra of CuO_x under eCO_2RR in 0.5 M CO_2-saturated $KHCO_3$ at -0.75 V using CA.

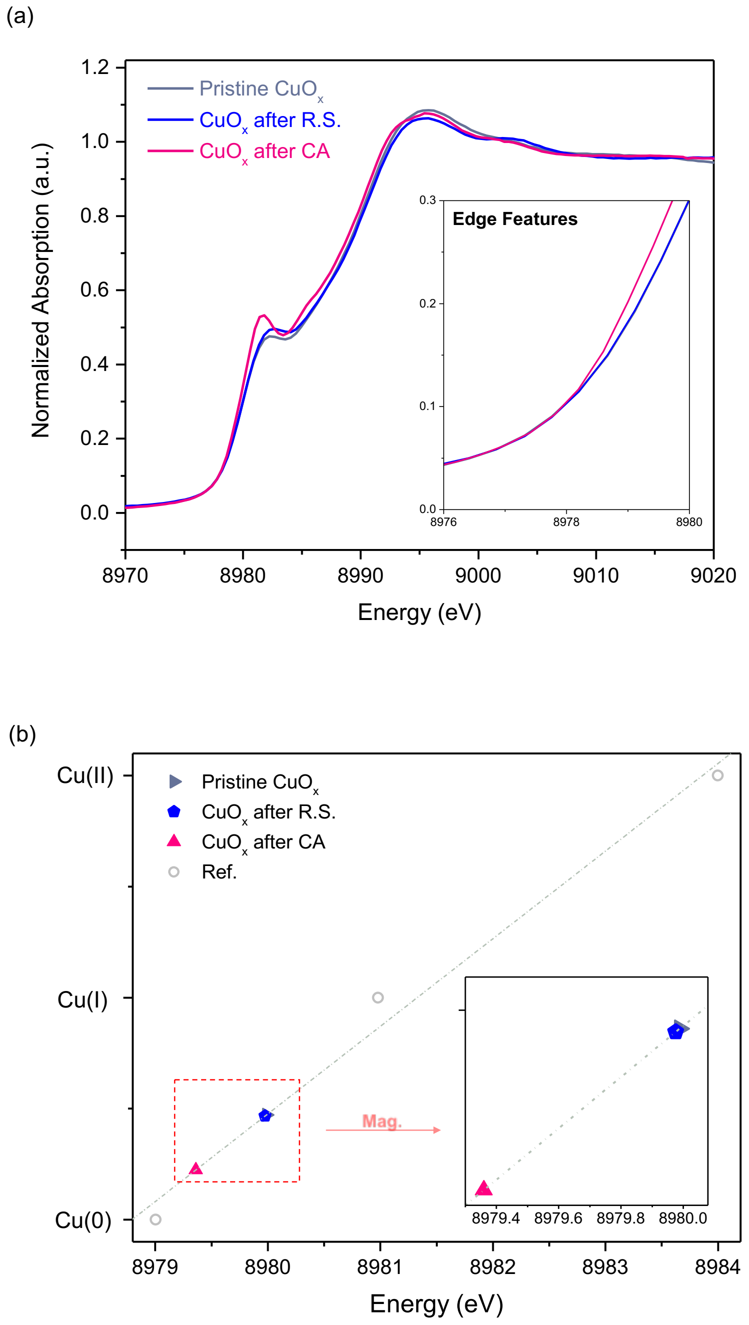

Supplementary Figure 10. (a) XANES spectra of pristine CuO_x, the CuO_x after a CO_2RR electrolysis using R.S., and the CuO_x after a CO_2RR electrolysis using CA. (b) Chemical nature of various Cu species – including commercial Cu, Cu_2O, and CuO as references, and the pristine CuO_x, the CuO_x after the R.S. CO_2RR treatment, and the CuO_x after the CA CO_2RR treatment. The working cathodic potential was -0.75 V, and the electrolyte was 0.5 M CO_2-saturated $KHCO_3$. Linear dependence ($R^2 = 0.99$) based on the reference samples is shown by the gray dash line.

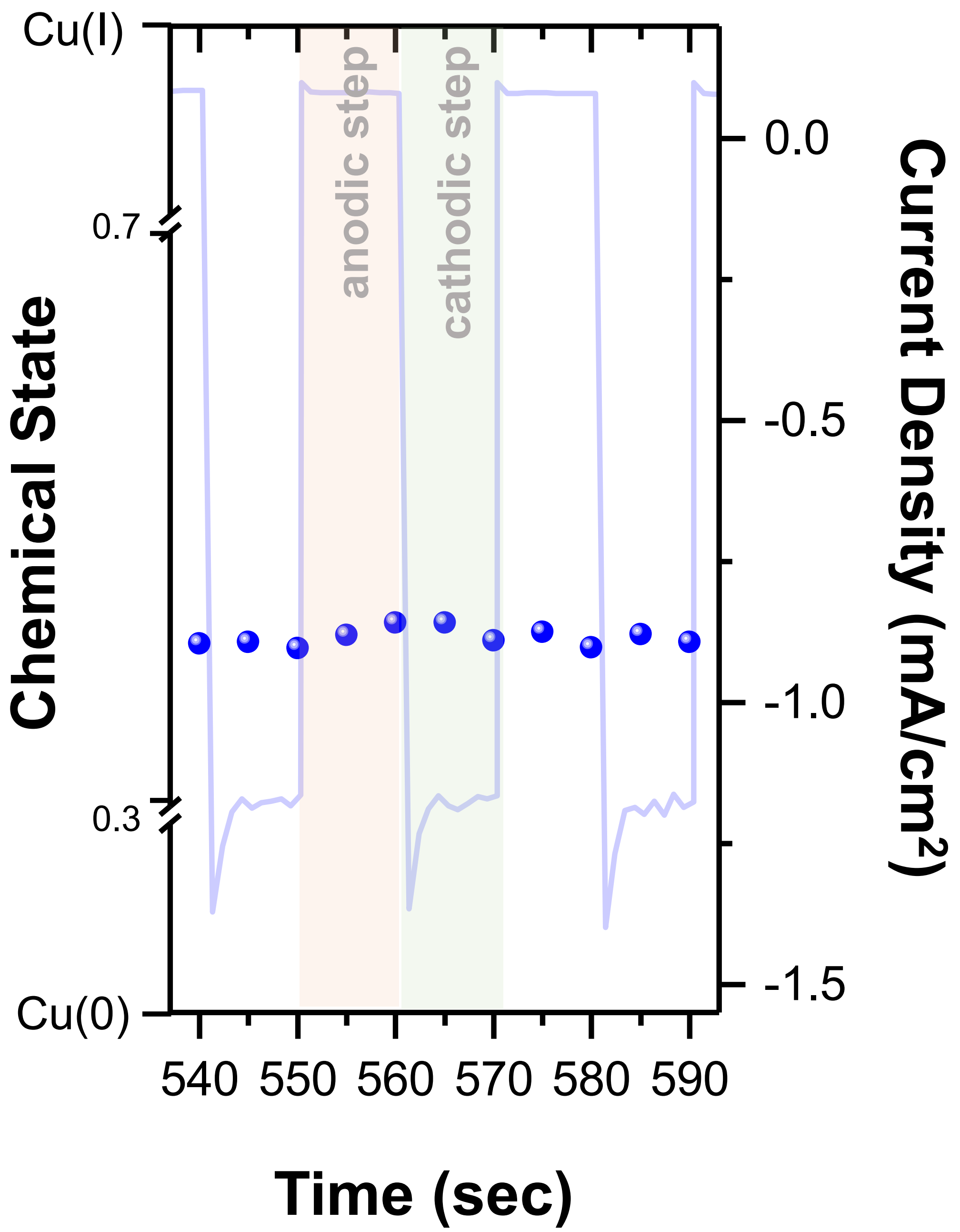

Supplementary Figure 11. Magnified portion of Figure 2b. The anodic step of R.S. was highlighted in orange, while the cathodic step was in green.

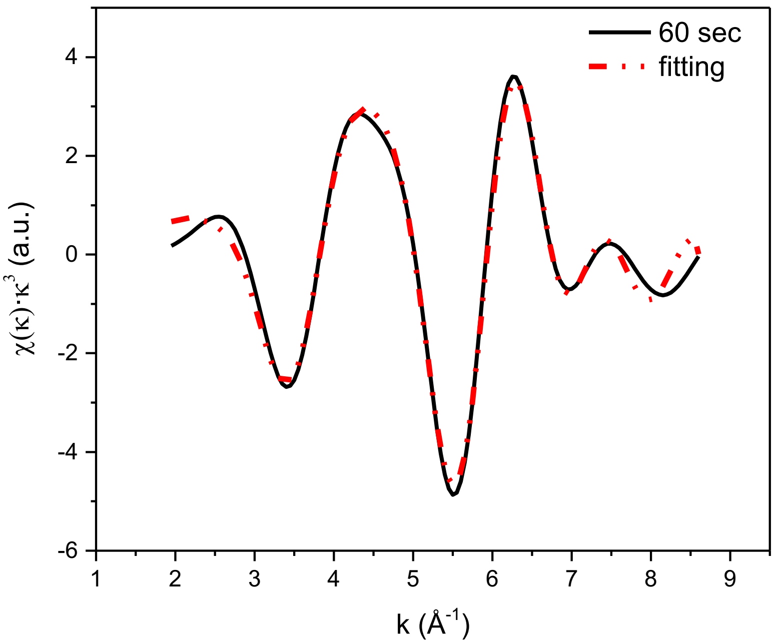

Supplementary Figure 12. Snapshot at 60 sec of time-resolved EXAFS fitting curves in k-space for CuO_x at -0.75 V in 0.5 M CO_2-saturated $KHCO_3$ using redox shuttle approach.

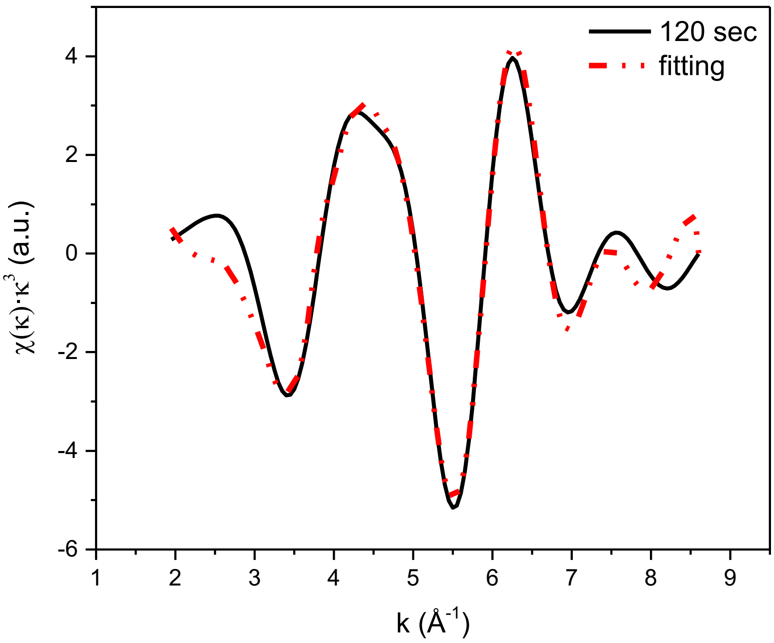

Supplementary Figure 13. Snapshot at 120 sec of time-resolved EXAFS fitting curves in k-space for CuO_x at -0.75 V in 0.5 M CO_2-saturated $KHCO_3$ using redox shuttle approach.

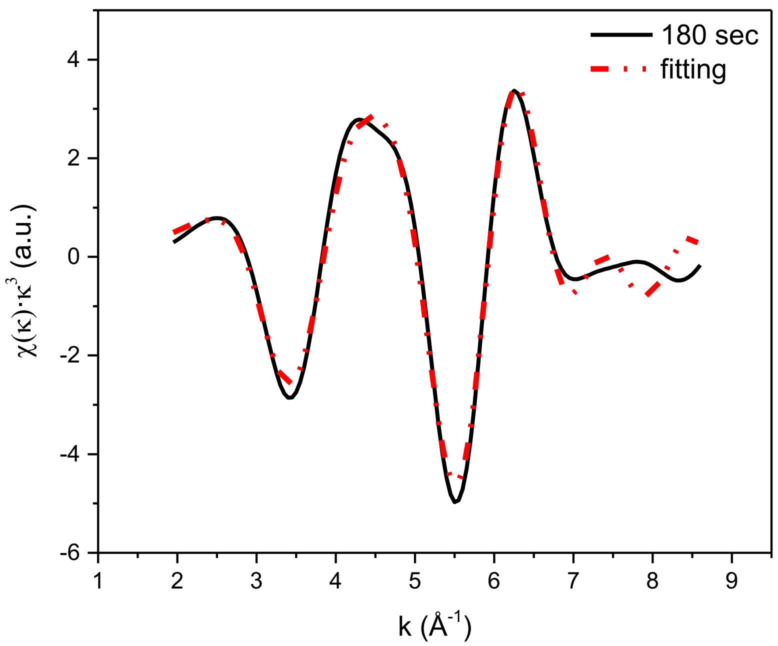

Supplementary Figure 14. Snapshot at 180 sec of time-resolved EXAFS fitting curves in k-space for CuO_x at -0.75 V in 0.5 M CO_2-saturated $KHCO_3$ using redox shuttle approach.

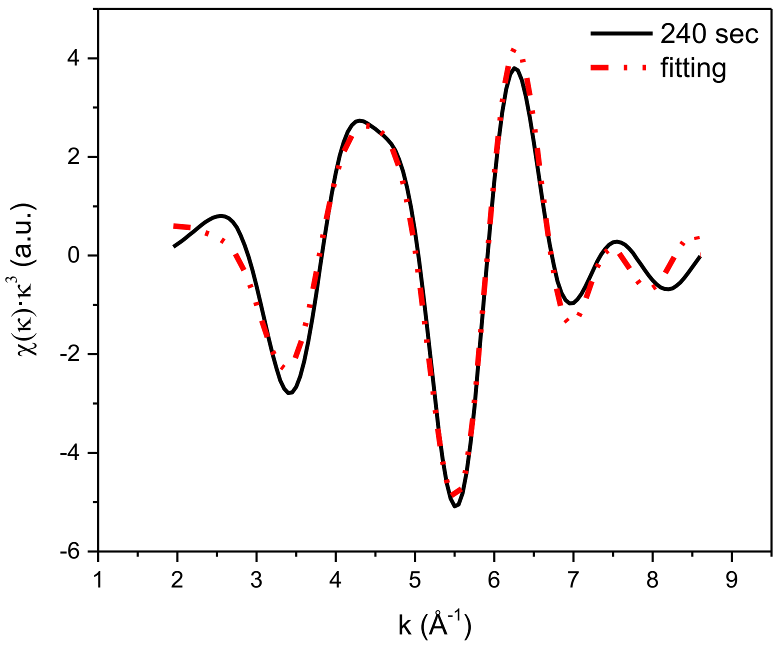

Supplementary Figure 15. Snapshot at 240 sec of time-resolved EXAFS fitting curves in k-space for CuO_x at -0.75 V in 0.5 M CO_2-saturated $KHCO_3$ using redox shuttle approach.

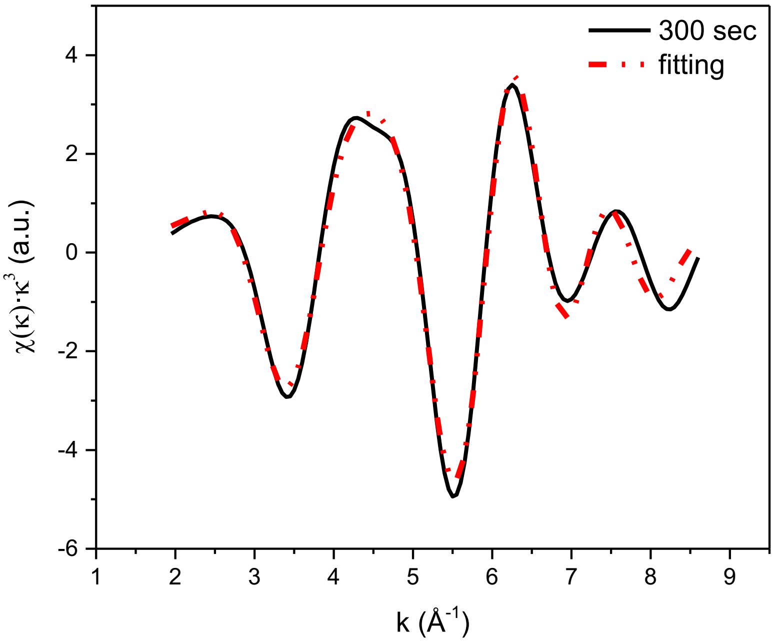

Supplementary Figure 16. Snapshot at 300 sec of time-resolved EXAFS fitting curves in k-space for CuO_x at -0.75 V in 0.5 M CO_2-saturated $KHCO_3$ using redox shuttle approach.

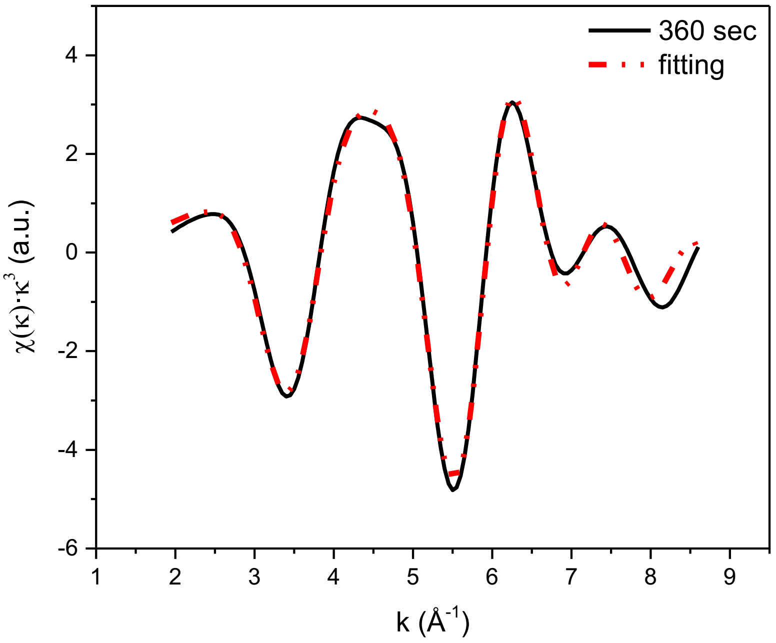

Supplementary Figure 17. Snapshot at 360 sec of time-resolved EXAFS fitting curves in k-space for CuO_x at -0.75 V in 0.5 M CO_2-saturated $KHCO_3$ using redox shuttle approach.

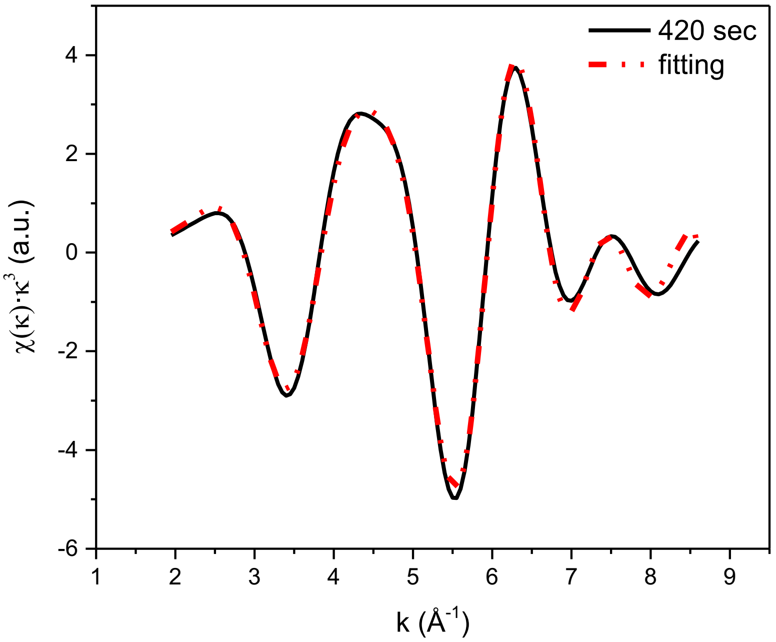

Supplementary Figure 18. Snapshot at 420 sec of time-resolved EXAFS fitting curves in k-space for CuO_x at -0.75 V in 0.5 M CO_2-saturated $KHCO_3$ using redox shuttle approach.

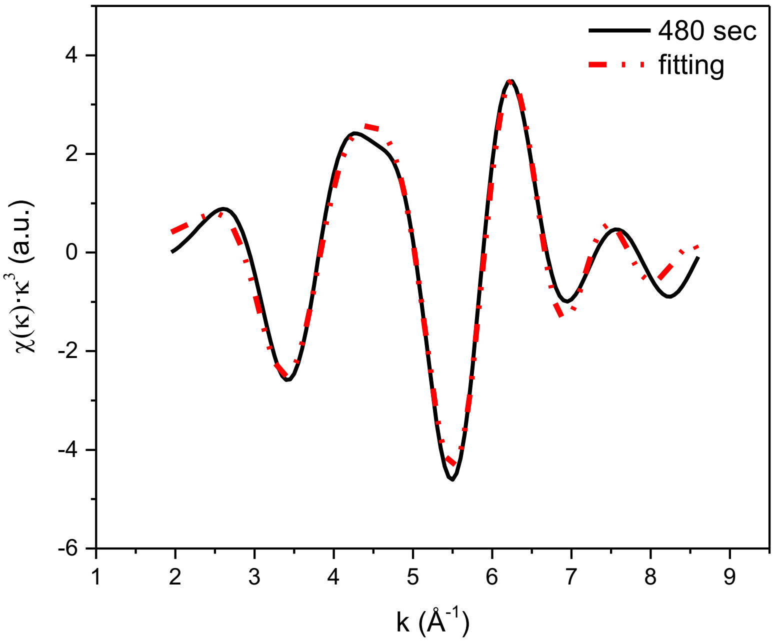

Supplementary Figure 19. Snapshot at 480 sec of time-resolved EXAFS fitting curves in k-space for CuO_x at -0.75 V in 0.5 M CO_2-saturated $KHCO_3$ using redox shuttle approach.

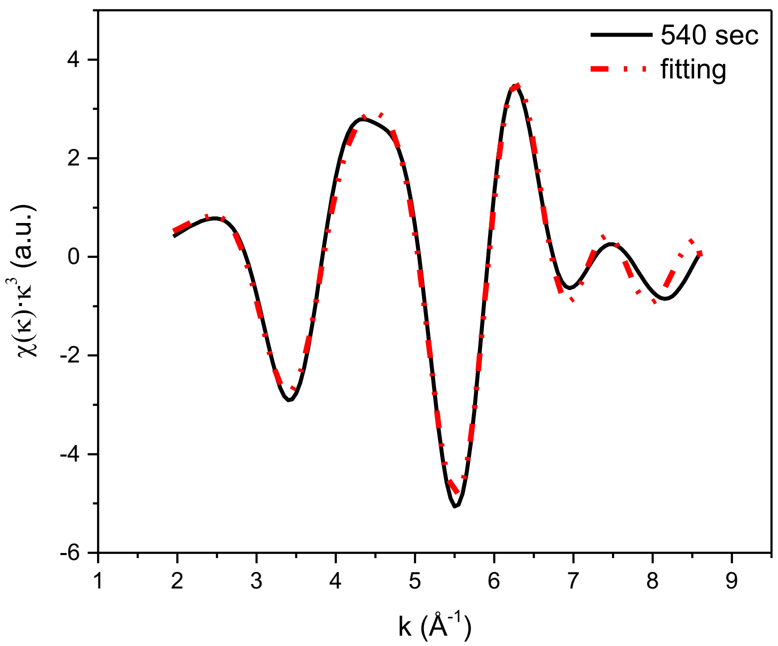

Supplementary Figure 20. Snapshot at 540 sec of time-resolved EXAFS fitting curves in k-space for CuO_x at -0.75 V in 0.5 M CO_2-saturated $KHCO_3$ using redox shuttle approach.

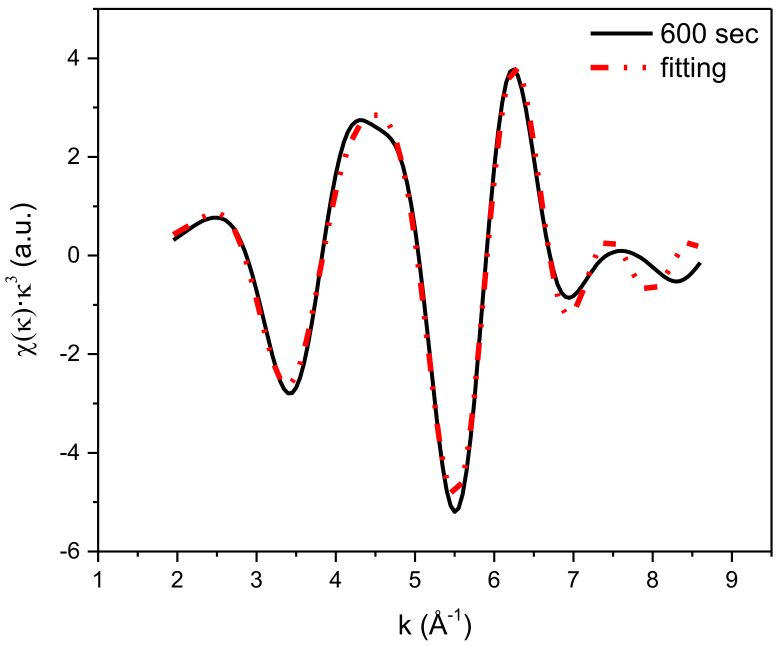

Supplementary Figure 21. Snapshot at 600 sec of time-resolved EXAFS fitting curves in k-space for CuO_x at -0.75 V in 0.5 M CO_2-saturated $KHCO_3$ using redox shuttle approach.

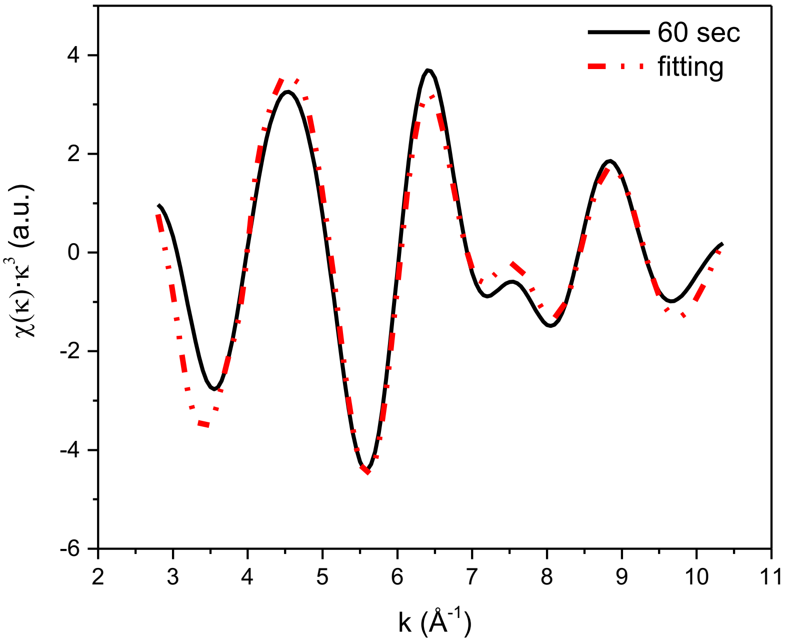

Supplementary Figure 22. Snapshot at 60 sec of time-resolved EXAFS fitting curves in k-space for CuO_x at -0.75 V in 0.5 M CO_2-saturated $KHCO_3$ using chronoamperometry.

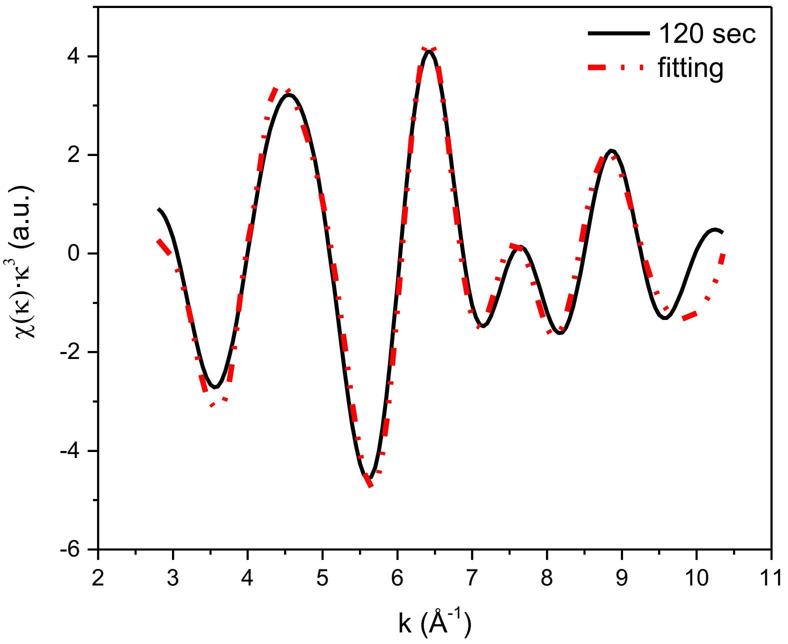

Supplementary Figure 23. Snapshot at 120 sec of time-resolved EXAFS fitting curves in k-space for CuO_x at -0.75 V in 0.5 M CO_2-saturated $KHCO_3$ using chronoamperometry.

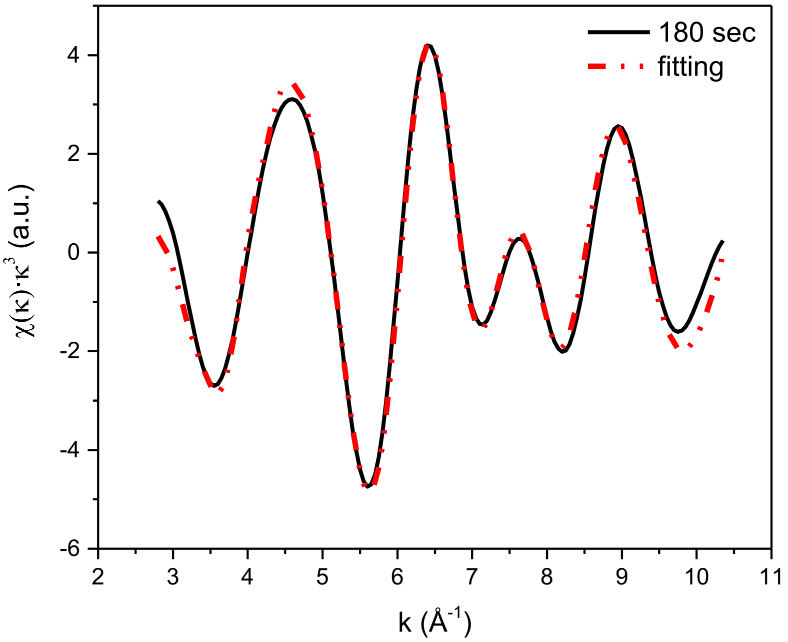

Supplementary Figure 24. Snapshot at 180 sec of time-resolved EXAFS fitting curves in k-space for CuO_x at -0.75 V in 0.5 M CO_2-saturated $KHCO_3$ using chronoamperometry.

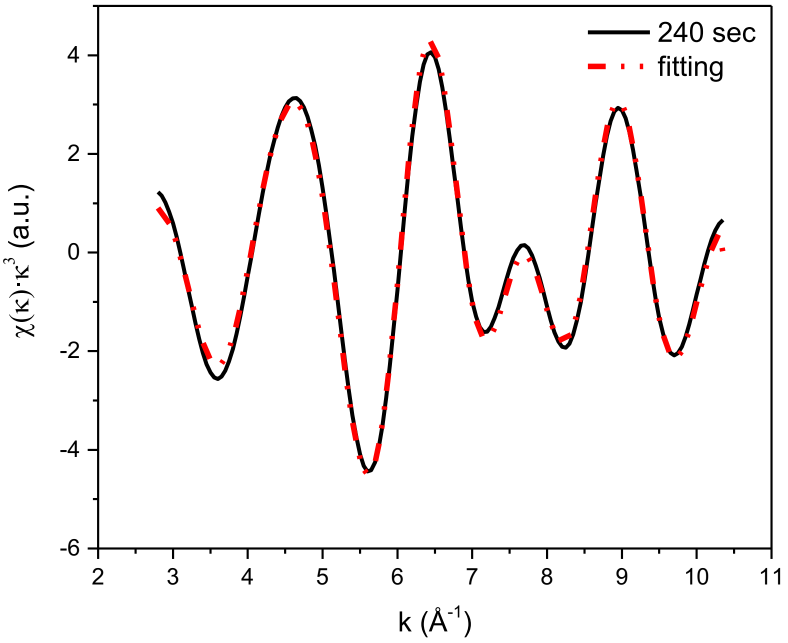

Supplementary Figure 25. Snapshot at 240 sec of time-resolved EXAFS fitting curves in k-space for CuO_x at -0.75 V in 0.5 M CO_2-saturated $KHCO_3$ using chronoamperometry.

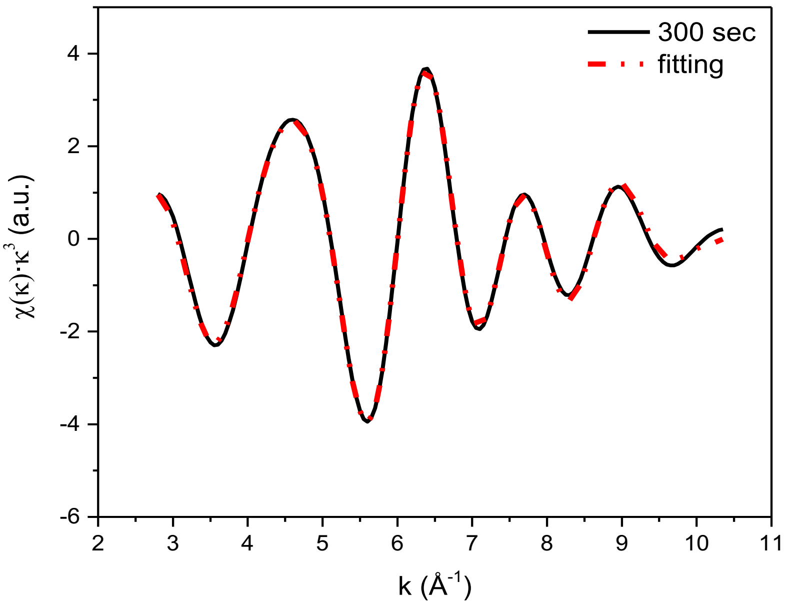

Supplementary Figure 26. Snapshot at 300 sec of time-resolved EXAFS fitting curves in k-space for CuO_x at -0.75 V in 0.5 M CO_2-saturated $KHCO_3$ using chronoamperometry.

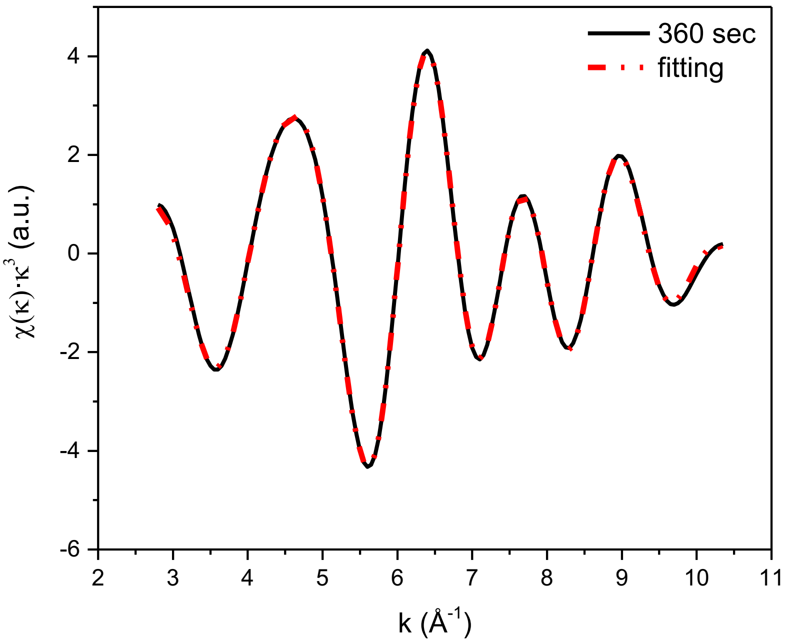

Supplementary Figure 27. Snapshot at 360 sec of time-resolved EXAFS fitting curves in k-space for CuO_x at -0.75 V in 0.5 M CO_2-saturated $KHCO_3$ using chronoamperometry.

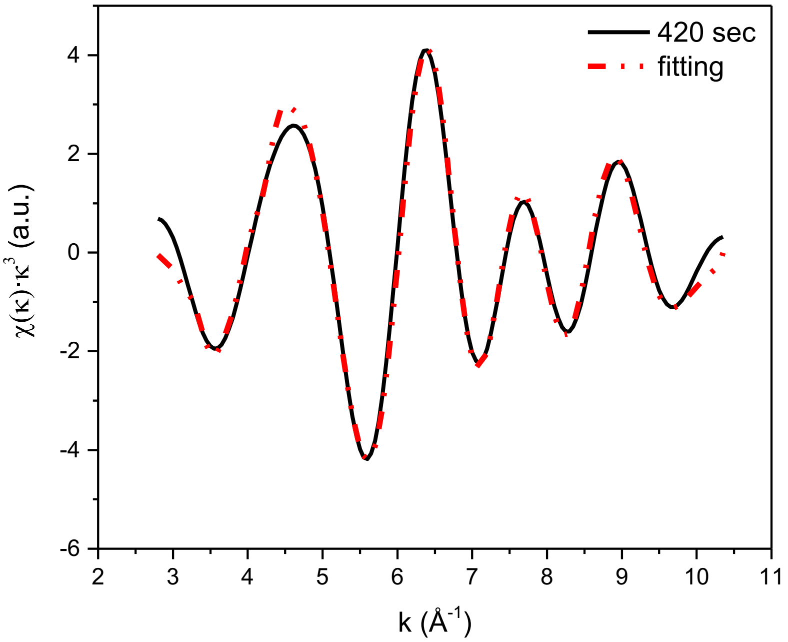

Supplementary Figure 28. Snapshot at 420 sec of time-resolved EXAFS fitting curves in k-space for CuO_x at -0.75 V in 0.5 M CO_2-saturated $KHCO_3$ using chronoamperometry.

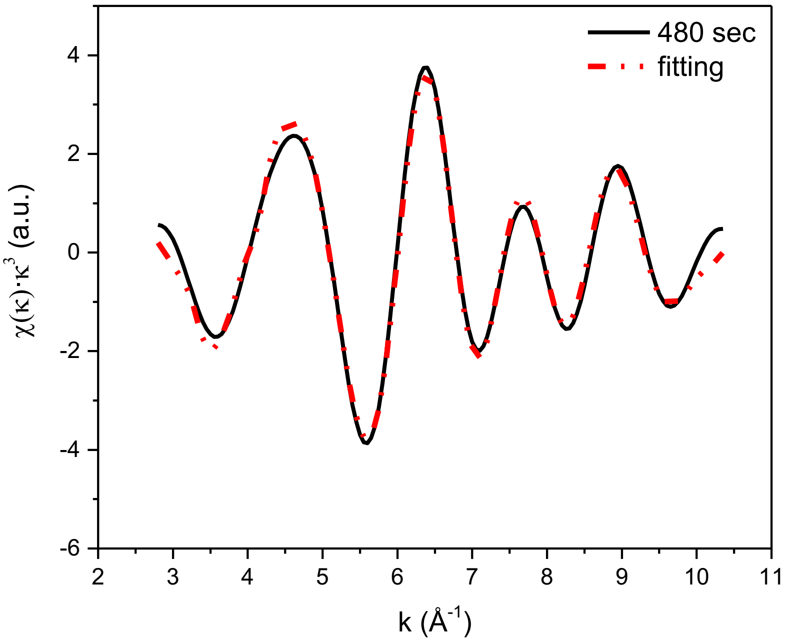

Supplementary Figure 29. Snapshot at 480 sec of time-resolved EXAFS fitting curves in k-space for CuO_x at -0.75 V in 0.5 M CO_2-saturated $KHCO_3$ using chronoamperometry.

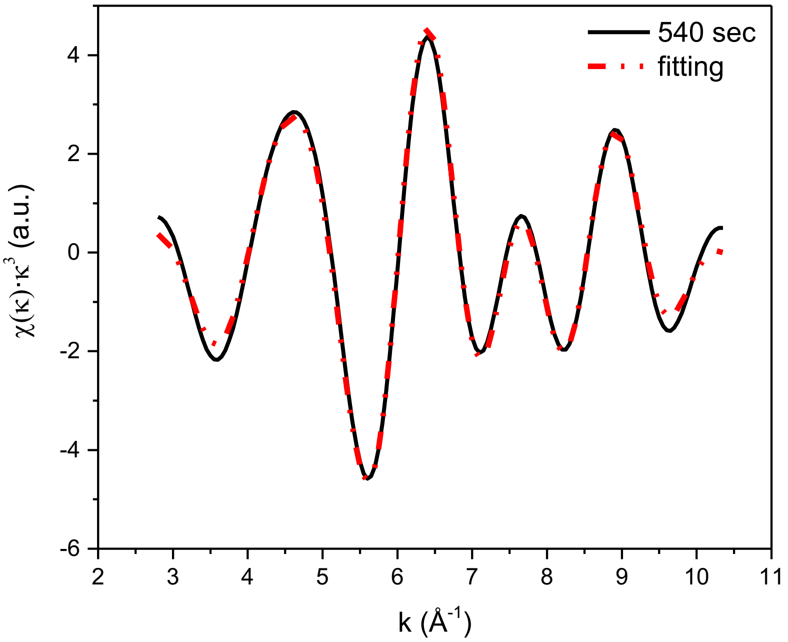

Supplementary Figure 30. Snapshot at 540 sec of time-resolved EXAFS fitting curves in k-space for CuO_x at -0.75 V in 0.5 M CO_2-saturated $KHCO_3$ using chronoamperometry.

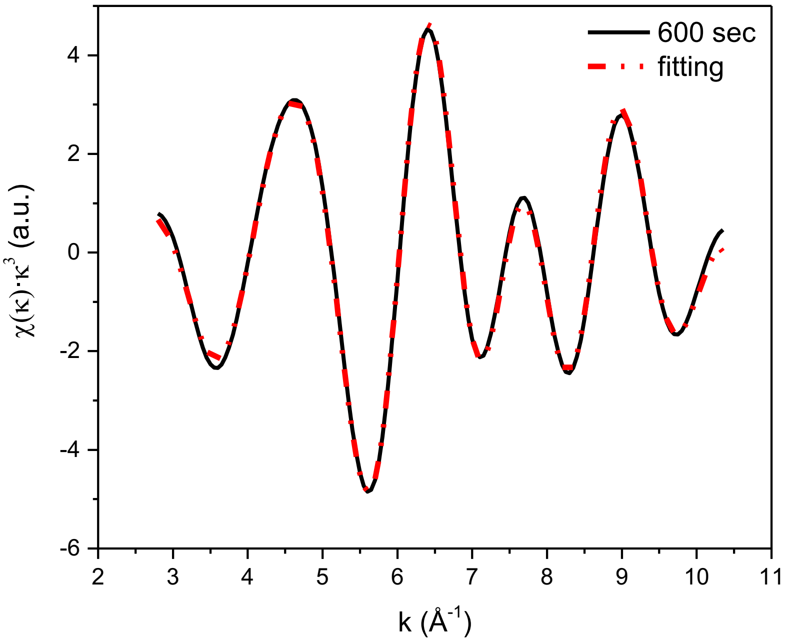

Supplementary Figure 31. Snapshot at 600 sec of time-resolved EXAFS fitting curves in k-space for CuO_x at -0.75 V in 0.5 M CO_2-saturated $KHCO_3$ using chronoamperometry.

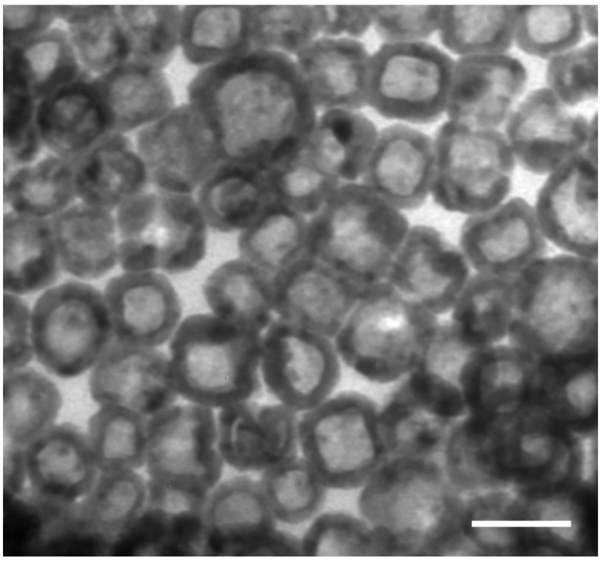

Supplementary Figure 32. TEM image of the CuO_x after 6-hours CO_2RR electrolysis. The electrochemical reaction was at -0.75 V in 0.5 M CO_2-saturated $KHCO_3$ using redox shuttle approach. Scale bar present in the image is 50 nm.

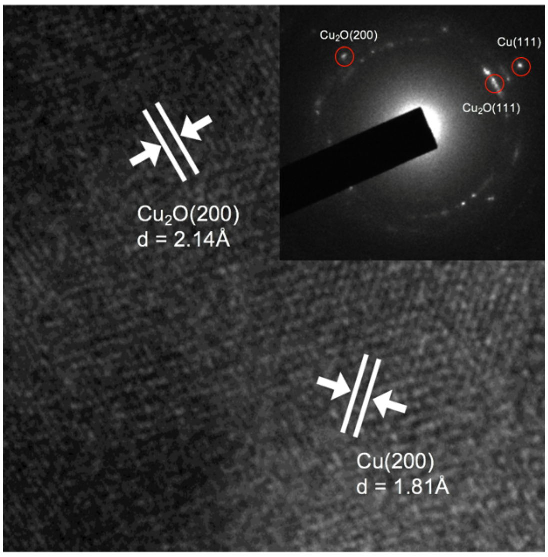

Supplementary Figure 33. HR-TEM image and SAED pattern (inset) of post-CuO_x. The CuO_x was used for a 6-hours eCO_2RR using redox shuttle approach in 0.5 M CO_2-saturated $KHCO_3$.

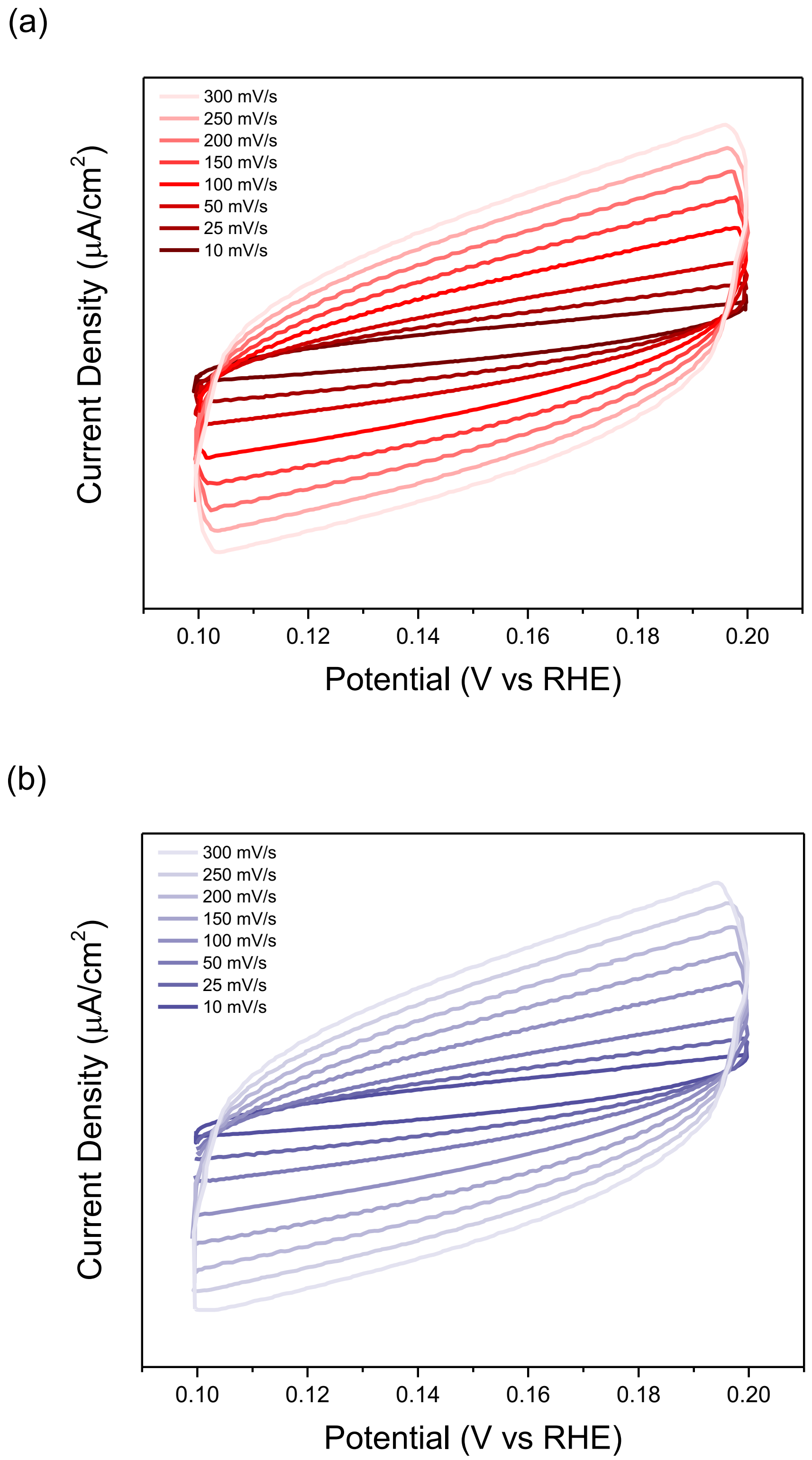

Supplementary Figure 34. Cyclic voltammograms in the region of 0.1-0.2 V for the CuO_x (a) before and (b) after CO_2RR treatment using Redox Shuttle (R.S.) at -0.75 V in 0.5 M N_2-saturated $KHCO_3$.

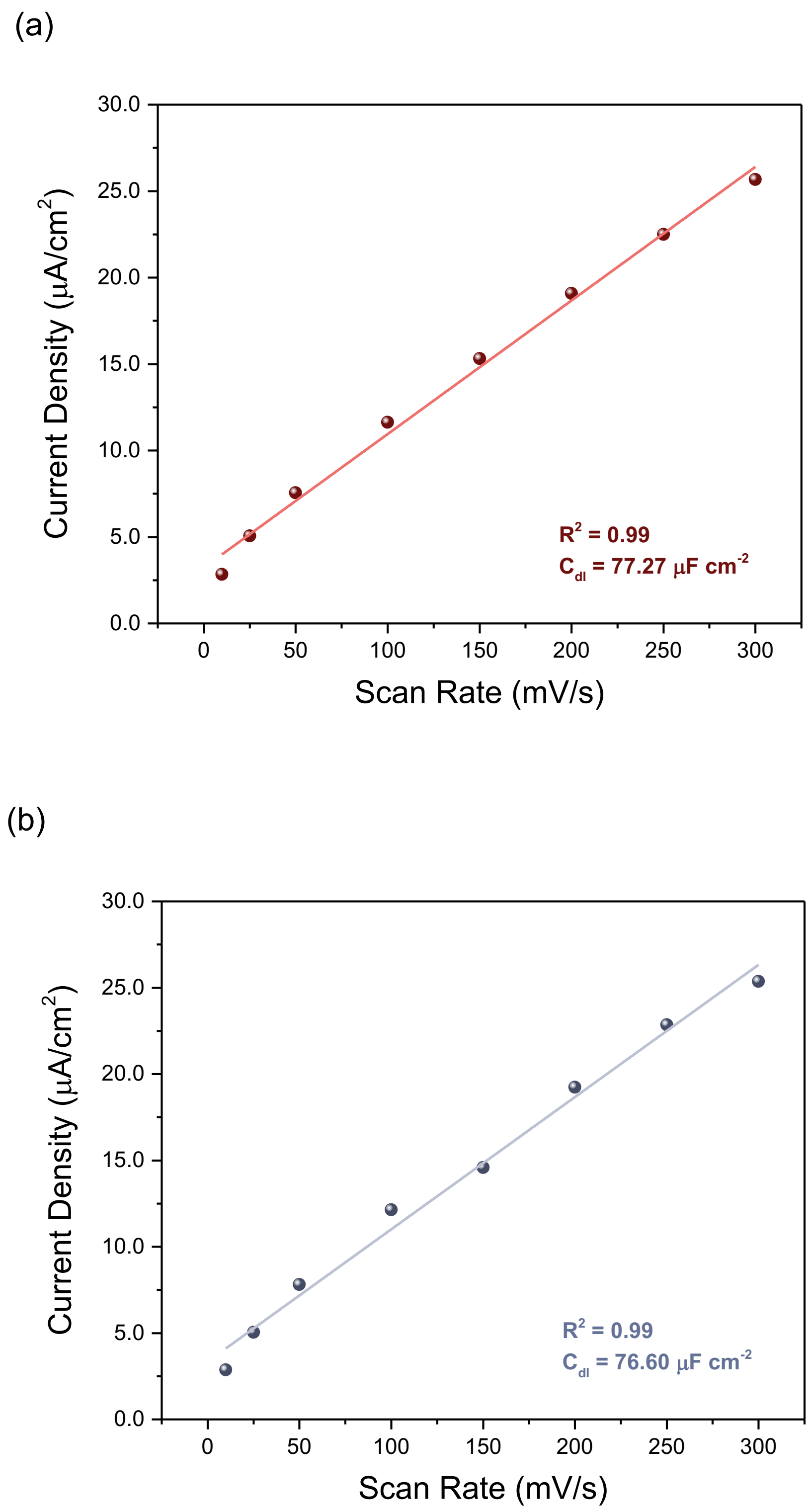

Supplementary Figure 35. Current density difference as a function of scan rate for the CuO_x (a) before and (b) after Redox Shuttle (R.S.) CO_2RR treatment at -0.75 V in 0.5 M N_2-saturated $KHCO_3$.

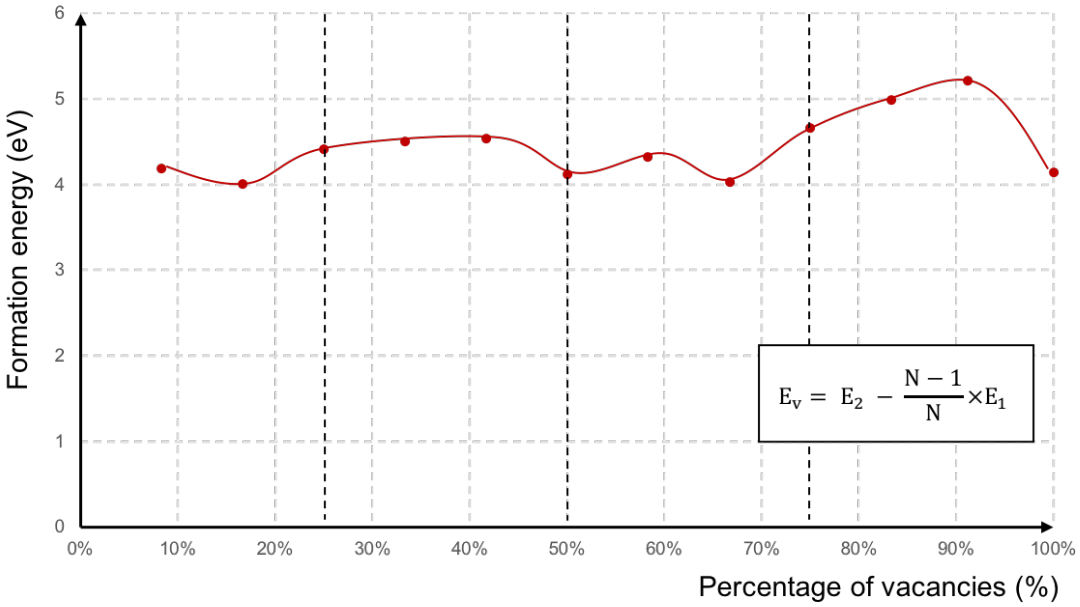

Supplementary Figure 36. The profile of the formation energy for various ratios of Cu and Cu(I).

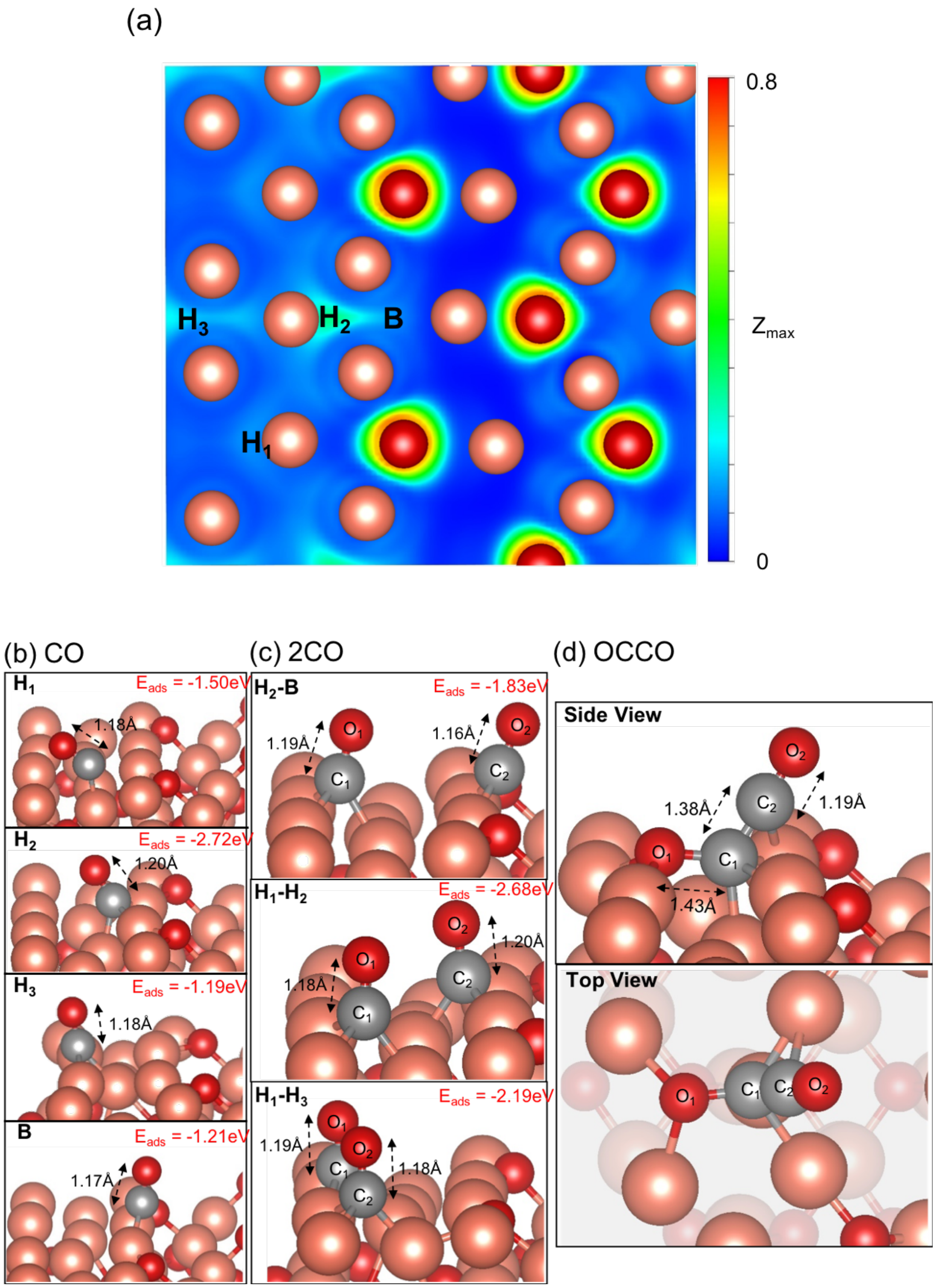

Supplementary Figure 37. (a) The electron-localization function plots of the ensemble. The schematic representations of the optimized structures of (b) CO, (c) 2CO and (d) OCCO on the ensemble. Surface reconstruction induced by CO adsorption-desorption perturbation has been considered for all surfaces.

Supplementary Note 1

We applied the CuO_x to conducting CO_2RR either using conventional chronoamperometry (CA) or using Redox Shuttle (R.S.) for 30 mins, and immediately toke CVs to investigate corresponding electrochemical oxidation responses (the procedure of the CVs the same as those in Figure 2). As depicted in Supplementary Fig. 7, the CV results showed that when the cycle time of R.S. was 20 sec (10 sec for reduction and 10 sec for oxidation), the oxidation responses between the applied potentials of -0.75 V and of -0.85 V were nearly the same, suggesting compositions of Cu species in both potentials were the same. Furthermore, when the applied potential went to a more negative one, namely -1.05 V, the corresponding electrochemical response was similar to those in -0.65 and in -0.85 V, despite the oxidation period increasing to 20 sec. On the contrary, using CA for 30 mins in -1.05 V (denoted as after), the oxidation amount of Cu species increased considerably, compared with that before CO_2RR. Overall, the results highly suggested R.S. enabled the composition of Cu species in CuO_x to be well preserved under CO_2RR, while CA did not.
